# Expansion within the CYP71D subfamily drives the heterocyclization of tanshinones synthesis in *Salvia miltiorrhiza*

**DOI:** 10.1038/s41467-021-20959-1

**Published:** 2021-01-29

**Authors:** Ying Ma, Guanghong Cui, Tong Chen, Xiaohui Ma, Ruishan Wang, Baolong Jin, Jian Yang, Liping Kang, Jinfu Tang, Changjiangsheng Lai, Yanan Wang, Yujun Zhao, Ye Shen, Wen Zeng, Reuben J. Peters, Xiaoquan Qi, Juan Guo, Luqi Huang

**Affiliations:** 1 State Key Laboratory Breeding Base of Dao-di Herbs, National Resource Center for Chinese Materia Medica, China Academy of Chinese Medical Sciences, Beijing, China; 2 College of Pharmaceutical Science, Yunnan University of Traditional Chinese Medicine, Kunming, China; 3 Roy J. Carver Dep. of Biochem., Biophys. & Mol. Biol., Iowa State University, Ames, IA USA; 4 Institute of Botany, the Chinese Academy of Sciences, Beijing, China

**Keywords:** Genomics, Plant evolution, Secondary metabolism

## Abstract

Tanshinones are the bioactive *nor*-diterpenoid constituents of the Chinese medicinal herb Danshen (*Salvia miltiorrhiza*). These groups of chemicals have the characteristic furan D-ring, which differentiates them from the phenolic abietane-type diterpenoids frequently found in the Lamiaceae family. However, how the 14,16-epoxy is formed has not been elucidated. Here, we report an improved genome assembly of Danshen using a highly homozygous genotype. We identify a cytochrome P450 (CYP71D) tandem gene array through gene expansion analysis. We show that CYP71D373 and CYP71D375 catalyze hydroxylation at carbon-16 (C16) and 14,16-ether (hetero)cyclization to form the D-ring, whereas CYP71D411 catalyzes upstream hydroxylation at C20. In addition, we discover a large biosynthetic gene cluster associated with tanshinone production. Collinearity analysis indicates a more specific origin of tanshinones in *Salvia* genus. It illustrates the evolutionary origin of abietane-type diterpenoids and those with a furan D-ring in Lamiaceae.

## Abstract (web summary)

Salvia miltiorrhiza is a medicinal plant that can produce the bioactive tanshinones. Here, the authors report the improved genome assembly and reveal the possible roles of three CYP71Ds in catalyzing the reactions leading to the formation of the characteristic furan D-ring of transhinones.

## Introduction

**Fig. 1:**
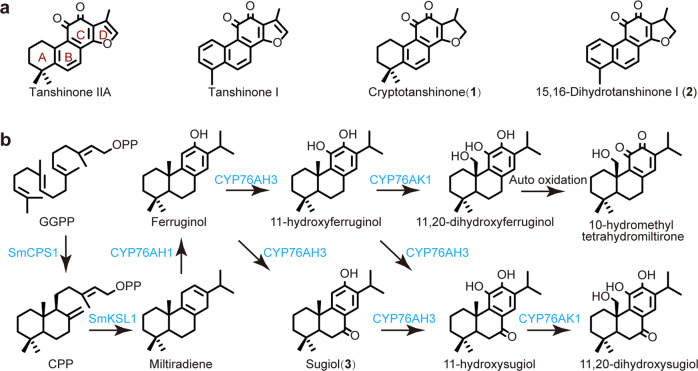
Tanshinones and partial biosynthetic pathway in Danshen. **a** Structures of the major tanshinone constituents of Danshen: tanshinone IIA, tanshinone I, cryptotanshinone (**1**) and 15,16-dihydrotanshinone I (**2**). **b** Elucidated steps for tanshinone biosynthesis in Danshen.

*Salvia miltiorrhiza* (Danshen in Chinese) is one of the oldest and most important traditional Chinese medicinal herbs^1^. Tanshinones are *nor*-diterpenoids that form the lipophilic bioactive constituents of Danshen (Fig. 1a)^2,3^. More specifically, these are phenolic abietane-type diterpenoids, which are widely found in the Lamiaceae family^4^. The tanshinones are uniquely characterized by the presence of a 14,16-ether D-ring, such as cryptotanshinone (**1**) and 15,16-dihydrotanshinone (**2**). However, this heterocycle is generally further oxidized to form a furan, as found in tanshinone I and tanshinone IIA. Tanshinones and chemically modified derivatives possess broad cardiovascular and cerebrovascular protective actions^5^. For example, the sodium sulfonate of tanshinone IIA is widely used in the clinic to treat patients with coronary artery disease^6^. Their pharmaceutical applications also include antioxidant, antibacterial, anti-inflammatory, antitumor, and anti-HIV activities^7^. Structure-activity relationship analysis indicates that the furan or dihydrofuran ring D structure influences pharmacological activities, thus highlighting the importance of D ring formation^8,9^.

Due to their medicinal properties, tanshinone biosynthesis has been intensively investigated for over a decade^10–13^. As labdane-related diterpenoids^14^, tanshinone biosynthesis is initiated by a class II diterpene cyclase; mainly the labdadienyl/copalyl diphosphate synthase SmCPS1, with subsequent further cyclization and rearrangement catalyzed by the class I diterpene synthase SmKSL1, which produces the abietane miltiradiene^12^. Three relevant cytochromes P450 (CYPs) also have been identified, CYP76AH1, CYP76AH3, and CYP76AK1, which catalyze hydroxylation at carbon-12 (C12)^13^, then C11 hydroxylation of the resulting ferruginol and, finally, C20 hydroxylation, respectively^11^. The promiscuity of these CYPs suggests that tanshinone biosynthesis may proceed via a metabolic network (Fig. 1b).

While CYPs prototypically catalyze hydroxylation, these monooxygenases are capable of mediating more complex transformations^15^. Among these, the formation of cyclic ethers is important due to the contribution of these structural features to biological activity. For example, of particular interest here is the formation of the characteristic furan D-ring, which is targeted for the generation of the sulfonated derivative of tanshinone IIA that is clinically relevant. However, the biosynthetic origin of this key distinguishing heterocycle in Danshen remains unknown.

Given the widespread production of phenolic abietane-type diterpenoids in the Lamiaceae family^4^, the addition of the D-ring differentiates the tanshinones. It also provides a key point of biosynthetic divergence. The formation of this cyclic ether is expected to be catalyzed by a CYP. However, CYPs form the largest enzymatic family in plants, comprising ~1% of all plant genes^16^. Not surprisingly, the sheer number of CYPs and the diversity of plant metabolism they operate in complicates the assignment of even basic metabolic function of CYPs on the basis of just phylogenetic relationship. Such functional attribution is difficult even within the more closely related CYP families or even subfamilies, which share >40% or >55% amino acid (aa) sequence identity, respectively. For example, members of the CYP71D subfamily function in indole alkaloid and flavonoid, as well as terpenoid biosynthesis^17^. Accordingly, CYPs readily undergo derivation of even basic metabolic function, further increasing the difficulty of identifying the relevant members of the superfamily.

Here, we assemble the genome of line bh2-7, which is derived from *S. miltiorrhiza* var. alba and bred to close to full homozygosity by successive selfings for six generations. Genome analyses reveal an expansion of the CYP71D subfamily. We identify possible roles for three CYP71Ds in catalyzing reactions leading to the formation of the characteristic furan D-ring of transhinones. Additionally, we discuss the evolutionary origin of tanshinones biosynthesis.

## Results

### Genome assembly and annotation

Danshen is highly heterozygous, which hindered genome assembly in previous sequencing efforts^18,19^. Hence, we selected line bh2-7 for sequencing. Originally derived from *Salvia miltiorrhiza* var. alba, line bh2-7 has been bred close to homozygosity by successive selfings for six generations (Supplementary Fig. 1), with an estimated heterozygosity of 0.43% based on 17-mer depth distribution using 26.73 Gb sequencing reads (Supplementary Fig. 2).

A total of 341.69 Gb of high-quality data were obtained using the Illumina Hiseq2000 platform, along with 30.13 Gb of data using the PacBio RS platform (6.46 kb read length in average) after reads filtering, representing approximately 542.36-fold and 50.21-fold coverage of the predicted Danshen genome (Supplementary Tables 1 and 2). The resulting assembly has a total length of 557 Mb with contig N50 of 505.21 kb and scaffold N50 of 1.26 Mb (Supplementary Table 3). These are approximately 2.7- or 93-fold longer than the previous assemblies of the Danshen genome^18,19^. The assembly covered about 89% of the genome according to the estimations derived from 17-mer depth distribution (623.58 Mb) (Supplementary Fig. 2) and flow cytometry (622 Mb) (Supplementary Fig. 3). Mapping the original short reads to the draft assembly indicates 97.87% overall coverage (Supplementary Table 4). BUSCO (Benchmarking Universal Single-Copy Orthologs) analysis and ESTs (Expressed sequence tags) mapping implied 91.10% and 99.56% genome completeness in terms of expected gene content, respectively (Supplementary Tables 5 and 6). In addition, 326,420 single nucleotide variations (SNVs) and 32,710 short indels were identified, corresponding to 0.64 SNVs per Kb. This heterozygosity value is 4.3-fold lower than the previous assembly of the Danshen genome^18^. All of the analyses indicate that this line bh2-7-based genome sequence has a relatively high-quality.

A total of 33,760 protein-coding genes with an average transcript length of 2771 bp were identified through the combination of ab initio, homology-based analyses, and RNA-Seq reads-assisted annotation (Supplementary Table 7). The number of annotated genes was similar to two previously reported Danshen genome assemblies, which reported 30,478^18^ and 34,598^19^ genes. About 81.97% of the genes have homologs in the TrEMBL protein database, and 67.83% can be functionally classified by InterPro. In summary, 83.13% of the genes have either known homologs and/or can be functionally classified (Supplementary Table 8). Regarding non-coding genes, we identified 129 microRNAs, 682 tRNAs, 844 small nuclear RNAs, and 282 rRNA fragments from the assembly (Supplementary Table 9). Repetitive elements accounted for 56.27% of the genome, of which about 46.30% are long terminal repeat (LTR) retrotransposons (Supplementary Table 10).

### Expansion of a clade within the CYP71D subfamily

**Fig. 2:**
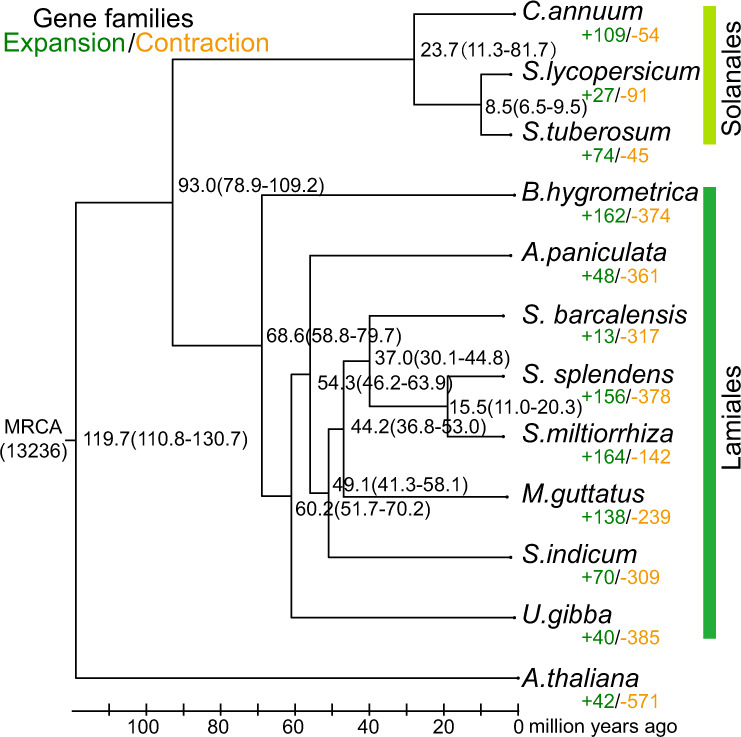
Phylogenetic analysis and divergence time estimations among 12 plant species. The tree was constructed based on 379 single-copy orthologous genes using the maximum likelihood method. Divergence times (Mya) are indicated in black numbers beside the branch nodes. The number of gene-family expansion and contraction events is indicated by green and orange numbers (respectively).

**Fig. 3:**
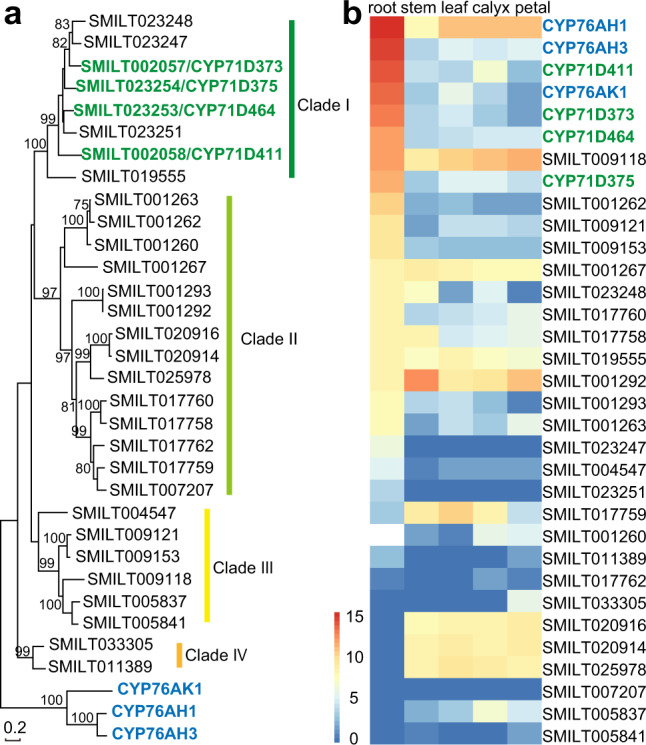
Phylogenetic relationship and expression profiles of CYP71D gene family in Danshen genome. **a** Phylogenetic analysis of Danshen CYP71D subfamily. Maximum likelihood method was used to construct the phylogenetic tree with 1000 replicate bootstrap support. The tree was rooted with the three CYP76 family members from Danshen previously shown to act in tanshinone biosynthesis. **b** Gene expression heatmap for the Danshen CYP71D subfamily members, as well as three CYP76 family members known to act in tanshinone biosynthesis, displaying relative expression level in root, stem, leaf, calyx, and petal of flowering plants. Source data underlying Fig. 3b are provided as a Source Data file.

To study expansion and contraction of the gene families, twelve plant species (*Salvia miltiorrhiza*, *Salvia splendens*, *Scutellaria barcalensis*, *Sesamum indicum*, *Andrographis paniculata*, *Mimulus guttatus*, *Boea hygrometrica*, *Utricularia gibba, Capsicum annuum*, *Solanum tuberosum, Solanum lycopersicum*, and *Arabidopsis thaliana*) were analyzed by CAFE (V2.1)^20^ (Fig. 2). The first eight species belong to Lamiales, while *C. annuum*, *S. tuberosum*, and *S. lycopersicum* belong to Solanales, which is closely related to Lamiales, and *A. thaliana* was used as an outgroup organism. Of these, only Danshen has been reported to have the ability to produce tanshinones. The analysis indicates that 164 gene families underwent significant expansion and 142 gene families underwent contraction in Danshen (Supplementary Data 1 and 2). The expanded families include CYPs, acyltransferases, laccases, auxin response factors, genes involving in biosynthesis of salvianolic acid (such as cinnamate 4-hydroxylase, rosmarinic acid synthase, 4-coumarate-CoA ligase). Among these, two expanded (sub)families, Plant_805 (in clade III in Fig. 3a) and Plant_13112 (in clade I of Fig. 3a), both fall within the CYP71D subfamily that has been reported to play role in terpenoid biosynthesis^17^.

In total, the Danshen genome contained 30 members of the CYP71D subfamily, which were further clustered into four clades (Fig. 3a). Gene structure analysis showed that most of the CYP71D genes in Danshen only have a single intron, consistent with the gene structure of other plant CYP71 clan^21^. Four genes in clade I (expansion family Plant_13112, *CYP71D373*, *CYP71D411*, *CYP71D464,* and *CYP71D375*) exhibited their greatest expression levels in roots, and showed similar expression profiles with *CYP76AH1*, *CYP76AH3*, and *CYP76AK1*, which are already known to play roles in tanshinone biosynthesis (Fig. 3b). Thus, these four CYP71Ds were considered to be potential candidate enzymes for tanshinone biosynthesis.

### RNAi indicates a role for the CYP71Ds in tanshinone biosynthesis

**Fig. 4:**
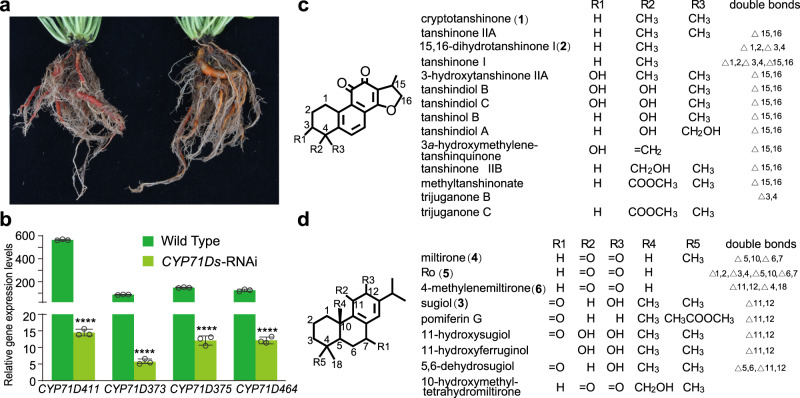
Phenotype, expression and metabolite profile changes of *CYP71Ds-*RNAi plants. **a** Coloration of roots from WT (left) and *CYP71Ds*-RNAi knockdown (right) plants. **b** Relative mRNA levels of the four CYP71D genes in *CYP71Ds*-RNAi and wild-type plants from transcriptome data. Error bars represent standard deviation SD (*n* = 3 biologically independent samples; ****P < 0.0001 by 2-sided Student’s *t* test). **c** Down-regulated tanshinone related diterpenoids in roots of *CYP71Ds*-RNAi relative to WT plants. **d** Up-regulated tanshinone related diterpenoids in roots of *CYP71Ds*-RNAi relative to WT plants. Source data underlying Fig. 4b are provided as a Source Data file.

Given that these CYP71Ds are closely related, an RNAi approach targeting a conserved region was utilized to knock down the expression of all four genes. In particular, as *CYP71D411* was the most highly expressed in roots, it was selected as the direct target (Fig. 3b). More precisely, a region of 453 bp (nucleotides 823-1276) was selected, as this exhibited >81% sequence identity with the three other targeted members of the clade, and was <71% identity to the next most closely related SMILT019555 (Supplementary Table 11). Global blast against all of the annotated genes reinforced this specificity; only genes from clade I in Fig. 3a have e-values < 1e^−5^ (Supplementary Table 12). The targeted fragment was cloned into the binary RNAi vector, pK7GWIWG (II) in an inverted-repeat fashion, and *Agrobacterium tumefaciens* was used to transfect Danshen to obtain transgenic plants. After four months of growth in the greenhouse, the *CYP71Ds*-RNAi plants exhibited a distinct orange phenotype in comparison with the wild-type (WT) root, which had the characteristic reddish color associated with tanshinones (Fig. 4a). There were no other obvious phenotypic differences.

To analyze the effect of this RNAi approach, RNA-Seq was carried out to compare the root transcriptomes of *CYP71Ds*-RNAi versus WT plants. As expected, the expression of the four targeted CYP71D subfamily members was significantly decreased in the *CYP71Ds*-RNAi lines, with their mRNA levels exhibiting a > 10-fold reduction compared with WT (Fig. 4b). Other members of the CYP71D subfamily showed trivial expression changes in the *CYP71Ds*-RNAi lines, except for *CYP71D414* (SMILT001293.1), which showed a > 6-fold expression increase (Supplementary Table 13). The expression of the five genes known to be involved in tanshinone biosynthesis, *CPS1*, *KSL1*, *CYP76AH1*, *CYP76AH3,* and *CYP76AK1*, also were reduced in *CYP71Ds*-RNAi lines (Supplementary Table 13). Beyond these genes, there were eight other genes down-regulated, and fifteen genes up-regulated in *CYP71Ds*-RNAi lines relative to WT.

### Metabolomic analysis suggests a role for CYP71Ds in heterocyclization

The different root colors illustrated a change in metabolic profile for the *CYP71Ds*-RNAi lines. To characterize this, metabolomic analysis of roots from both *CYP71Ds*-RNAi and WT plants was carried out using LC-electrospray ionization-qTOF-MS and GC-electron impact (EI)-triple quadrupole (QqQ)-MS methods^22^. The LC-qTOF-MS analysis revealed that 24 metabolites showed significantly reduced and 16 metabolites showed significantly elevated accumulation in *CYP71Ds*-RNAi lines compared with WT (Fig. 4c, d; Supplementary Data 3 and 4). The more targeted GC-MS analysis revealed that three known tanshinone biosynthetic intermediates, miltiradiene, abietatriene, and ferruginol, accumulated at higher levels in the *CYP71Ds*-RNAi lines (Supplementary Fig. 4).

A total of 21 out of the 24 metabolites with reduced accumulation in the *CYP71Ds*-RNAi lines could be identified (Supplementary Data 3). Notably, 14 of these metabolites contain a 14,16-epoxy D-ring (Fig. 4c). For example, the levels of active compounds of Danshen including cryptotanshinone (**1**) and 15,16-dihydrotanshinone I (**2**), and tanshinone IIA were decreased ~27, ~2, and ~3-fold, respectively. This indicated that the reduced expression of these four CYP71D subfamily members seems to decrease the formation of the characteristic heterocyclic D-ring in the tanshinones. Accordingly, we hypothesized that at least one of the down-regulated CYP71Ds play a role in the heterocyclization required for tanshinone biosynthesis.

Integrating the LC-MS and GC-MS analyses, there were a total of 19 metabolites found to exhibit elevated accumulation in the *CYP71Ds*-RNAi lines. Nine are known or potential intermediates in tanshinone biosynthesis (Fig. 4d and Supplementary Data 4). These included sugiol (**3**), 11-hydroxysugiol, miltirone (**4**) and 10-hydroxymethyl tetrahydromiltirone. Of particular interest, **4** has been predicted to be a key intermediate, as the immediate precursor of **1** and subsequently derived tanshinone IIA, and accumulated in ~9-fold higher amounts in *CYP71Ds*-RNAi lines relative to WT. In addition, increases were observed with Ro (**5**), as well as 4-methylenemiltirone (**6**), which similarly do not contain the heterocyclic D-ring, with the content of **5** and **6** increased ~38 and ~3-fold in *CYP71Ds*-RNAi relative to WT plants. Given together, we hypothesized that some of the accumulated diterpenoids in the *CYP71Ds*-RNAi plants might serve as substrates for these CYPs, particularly **4**, **5**, and **6**, which appear to be poised for D-ring heterocyclization.

### Biochemical analysis of the targeted CYP71D clade

To investigate the biochemical activity of these CYPs, recombinant expression in yeast (*Saccharomyces cerevisiae*) was employed, as this has proven to be successful for previous such characterization^11,13^. For this purpose, full-length cDNAs for all four CYP71Ds (*CYP71D373*, *CYP71D375*, *CYP71D411,* and *CYP71D464*) were cloned into the pESC-His expression vector, and the resulting constructs transformed into the WAT11 yeast strain in which the endogenous NADPH-CYP reductase has been replaced by one from *A. thaliana*^23^. In vitro assays were then carried out with microsomal preparations from induced cultures of this recombinant yeast, using **4**, **5**, and **6** as potential substrates.

**Fig. 5:**
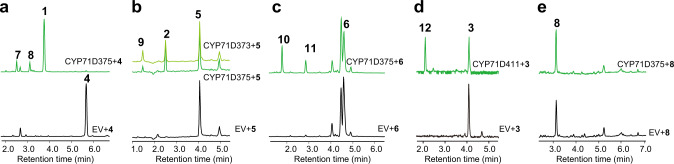
Catalytic activity of targeted CYP71D subfamily members with putative tanshinone intermediates accumulating in *CYP71Ds*-RNAi plant roots. Extracted ion chromatograms showing the in vitro catalytic activity of (**a**) CYP71D375 with **4**. **b** CYP71D373 and CYP71D375 with **5**. **c** CYP71D375 with **6**. **d** CYP71D411 with **3**. **e** CYP71D375 with **8**. In each case, enzyme-mediated activity is indicated by the green chromatograms (with the relevant enzyme noted), while those for the empty vector (EV) negative control assays are in black.

Only CYP71D375 accepts miltirone (**4**) as a substrate, with three products detected (Fig. 5a). The major product was found to reflect mass addition of [M] = 13.9789 Da and was identified as the heterocyclic **1** by comparison to an authentic standard, and is labeled as such here – i.e., **1** (Supplementary Fig. 5a). Two minor products also were observed, with the earlier eluting product (**7**) found to have a mass addition of [M] = 15.9926 Da, suggesting this was generated by a hydroxylation reaction (Supplementary Fig. 5b). This compound also was detected in Danshen roots, and was determined to be 16-hydroxymiltirone (**7**) by NMR (Supplementary Fig. 6). The remaining minor product (**8**) was found to have a mass addition of [M] = 31.9894 Da, and was identified as the known 14,16-dihydroxy derivative neocryptotanshinone (**8**) by comparison to an authentic standard (Supplementary Fig. 5c). These results indicate that CYP71D375 functions to convert **4** to the 14,16-epoxy heterocyclic derivative **1**, which is consistent with the accumulation of **4** in the *CYP71Ds*-RNAi lines.

When **5** was used as substrate, two products were observed with both CYP71D373 and CYP71D375 (Fig. 5b). The major product was found to have a mass addition of [M] = 13.9797 Da, identified as the heterocyclic derivative **2** by comparison to an authentic standard (Supplementary Fig. 7a). Similarly, the mass of the minor product suggests that it was generated by hydroxylation, and was identified as the 16-hydroxy derivative (**9**) by comparison to an authentic standard (Supplementary Fig. 7b).

Only CYP71D375 accepts **6** as a substrate, with two products detected (Fig. 5c). The structure of **10** was inferred based on various sources of information including MS analysis, retention time, compound degradation behavior, known CYP71D375-catalyzed chemistry and chemical logic. On this basis, **10** is speculated to be 16-hydroxy-4-methylenemiltirone. **11** was identified as methylenedihydro-tanshinquinone by comparison to an authentic standard (Supplementary Fig. 7c–d).

The biochemical assays with **4**, **5**, and **6** indicated that CYP71D411 and CYP71D464 are not involved in heterocyclization. Conversely, while other compounds that accumulated in RNAi lines also were tested with these four CYP71D subfamily members, no products were detected with CYP71D373, CYP71D375, or CYP71D464. However, CYP71D411 accepts sugiol (**3**) as a substrate, and the mass [M-H]^-^ = 315.1947 of the product was indicative of hydroxylation (Fig. 5d). In order to characterize this product, the enzymatic reaction system was scaled-up to 100 mL to enable purification of sufficient amounts for structural analysis by NMR, which identified this as 20-hydroxysugiol (**12**) (Supplementary Figs. 8–9).

**Fig. 6:**
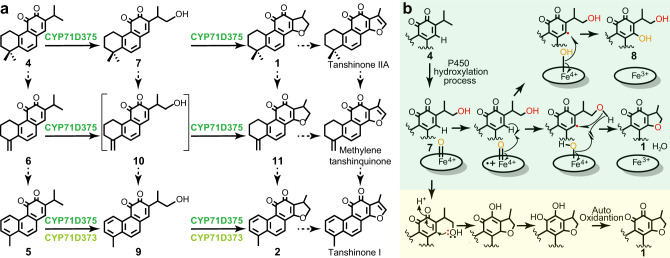
Catalytic process analysis of CYP71D373 and CYP71D375. **a** Role of CYP71D373 and CYP71D375 in forming the characteristic tanshinone D-ring heterocycle in a metabolic grid for tanshinone biosynthesis in Danshen. **b** Proposed reaction mechanisms for heterocyclization of **4** to **1**.

Altogether, these in vitro assays provided biochemical evidence for specific functions of three members of the targeted CYP71D clade in tanshinone biosynthesis. In particular, the results suggest that CYP71D375 and CYP71D373 are important for heterocyclization to form the characteristic D-ring of the tanshinones (Fig. 6a), while CYP71D411 acts as a C20 hydroxylase of **3**.

### Biochemical analysis of heterocyclization of miltirone by CYP71D375

The heterocyclization catalyzed by CYP71D375 represents the characteristic step in tanshinone biosynthesis (Fig. 6a). To probe the biochemical mechanism underlying the formation of this cyclic ether, the conversion of **4** to **1** was further investigated here. The presence of the 14,16-dihydroxylated derivative **8** might suggest that this serves as an intermediate, such that heterocyclization occurs via dehydration. However, when **8** was fed to CYP71D375, product **1** was not observed (Fig. 5e), indicating that heterocyclization is not achieved by dehydration. Accordingly, it seems most likely that CYP71D375 catalyzes cyclic ether formation directly from the 16-hydroxylated derivative **7**, which we hypothesize utilizes the basic CYP free radical mechanism. Accordingly, following initial hydroxylation of **4** to **7**, CYP71D375 would mediate the prototypical hydrogen abstraction from C14, but the resulting radical would then undergo direct (hetero)cyclization to form **1** (Fig. 6b). However, we cannot rule out alternative mechanisms, such as Michael addition or ketone formation (Supplementary Fig. 10), although we did not find any peaks with molecular weights corresponding to possible intermediates from such mechanisms.

### Mutational analysis of heterocyclization of miltirone by CYP71D375

**Fig. 7:**
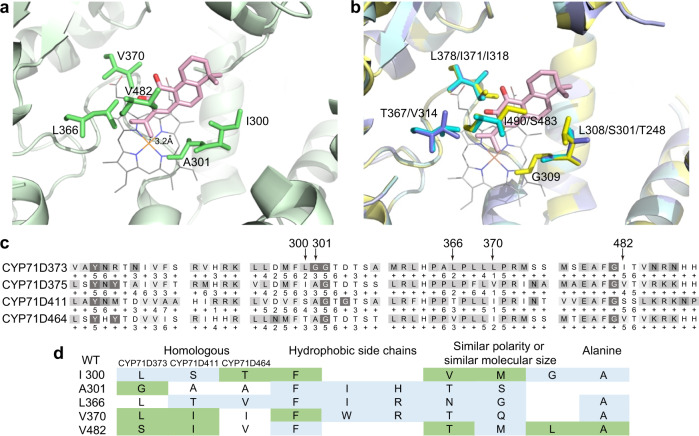
Docking analysis and mutation verification of CYP71Ds active sites in catalyzing miltirone. **a** Docking result for miltirone (pink) in the CYP71D375 model, with side-chains for the targeted residues shown (green). **b** Docking result for miltirone, with side-chains of the targeted residues shown (as indicated), in the models for CYP71D373 (yellow), CYP71D411(blue) and CYP71D464 (purple). **c** Alignment of the regions around the five proposed distinguishing active site residues. **d** Table indicating positive (green) and negative (blue) impact of CYP71D375 mutations on enzymatic reaction with miltirone.

To examine the enzymatic structure-function relationships underlying the heterocyclase activity of CYP71D375, we used protein modeling with substrate docking to guide site-directed mutagenesis. Models of all four CYP71Ds examined here were generated based on the crystal structure reported for CYP76AH1^24^. Miltirone (**4**) was then docked into these CYP71D models, enabling estimation of the proximity of amino acids lining the active site to **4** (Fig. 7a, b). Of particular interest were such residues (within 5 Å of **4**) that substantially differ between CYP71D375, as well as CYP71D373, which can both catalyze heterocyclization, versus CYP71D411 and CYP71D464. From this, five residues were targeted for mutagenesis (Fig. 7c).

These residues were subjected to a series of substitutions in CYP71D375, with a total of 37 mutants constructed, and the effect of these on catalytic function with **4** examined (Fig. 7d). The results indicated that L366 is a key residue, as all mutants at this position lost the ability to react with **4**. Notably, L366 is situated 5 residues after the ExxR-motif in substrate recognition site 5, a position which has been proposed to direct substrate-heme interaction in CYPs^25^. Similarly, for A301, a position that also has been suggested to affect substrate-heme interactions^26^, only substitution of the smaller glycine for A301 retained catalytic activity.

### Gene clustering contributed to evolution of the tanshinone pathway

The results reported above further increase the number of genes associated with tanshinone biosynthesis, with three diterpene synthases, *SmCPS1*, *SmCPS2,* and *SmKSL1*^12,22^, and now six CYPs, *CYP76AH1*, *CYP76AH3*, *CYP76AK1*, *CYP71D373*, *CYP71D375,* and *CYP71D411*^11,13^. These nine genes were distributed over five scaffolds (Supplementary Fig. 11). It has previously been reported that *SmCPS2* is co-clustered with *CYP76AH1* and *CYP76AH3*, demonstrating that Danshen contains a biosynthetic gene cluster for tanshinone production^18^. In order to further explore the role of gene clustering in the evolution of tanshinone biosynthesis, Hi-C was employed to assign the assembled scaffolds to chromosome-scale pseudomolecules. Altogether, 1115 scaffolds were anchored to 340 super-scaffolds with N50 of ~73.9 Mb. While falling short of chromosomal definition, we obtained one pseudochromosome (~65 Mb) with remarkably higher inner fragment interactions, defined here as pseudochromosome 6 (Supplementary Fig. 12). This pseudochromosome 6 includes all of the identified genes involved in tanshinone biosynthesis except *CYP76AK1* (Supplementary Fig. 13).

Notably, *SmCPS1*, *SmCPS2*, *SmKSL1*, *CYP76AH1,* and *CYP76AH3* are clustered within a 310 kb region (Supplementary Fig. 13), defining an even larger tanshinone biosynthetic gene cluster in Danshen. This cluster contains a number of potential gene duplicates. For example, *SmCPS1* and *SmCPS2* are the two most closely related class II diterpene cyclases in Danshen, with *SmCPS2* predicted to be involved in tanshinone biosynthesis in aerial tissues, while *SmCPS1* is known to play a role in root tanshinone biosynthesis^22,27^. Similarly, although CYP76AH1 and CYP76AH3 catalyze distinct reactions in tanshinone biosynthesis, these are also quite closely related to each other^11,13^. In addition, the four CYP71Ds investigated here are also found within a ~160 kb region (Supplementary Fig. 13). To examine the origins of these two gene clusters, we analyzed the collinearity of these two regions from Danshen with *S. splendens, S. barcalensis* and *S. indicum*, which have relatively high-quality genome sequences^28–30^. This comparison showed that the Danshen tanshinone biosynthetic gene cluster exhibits some collinearity with all three of these related species. By contrast, the Danshen CYP71D subfamily gene cluster only exhibits evident collinearity with *S. splendens*, while the orthologous loci in *S. barcalensis* does not contain any members of this subfamily (Supplementary Fig. 14). This then provides an opportunity to investigate the mechanism of diterpenoid diversification in Lamiaceae.

**Fig. 8:**
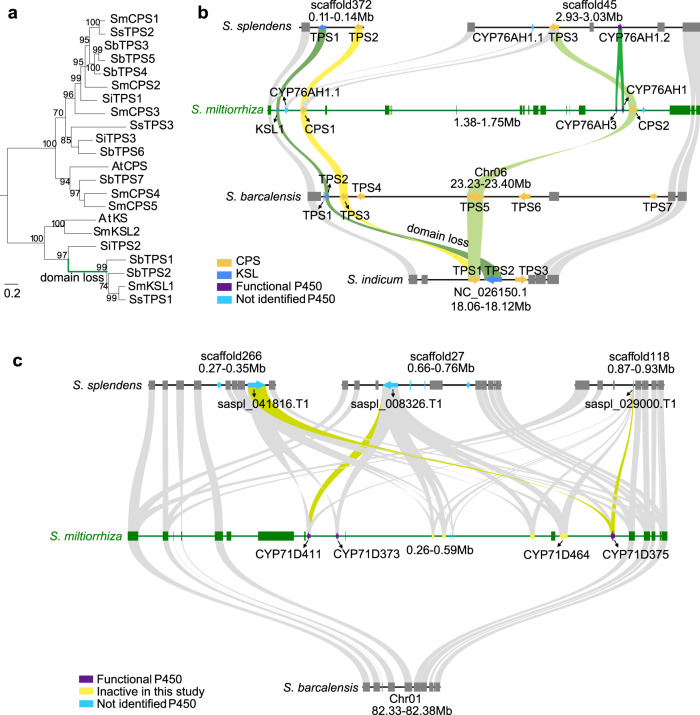
Tandem duplication and syntenic analysis of genes involved in tanshinone biosynthesis. **a** ML phylogeney of diterpene cyclases and synthases from *S. miltiorrhiza* (Sm), *S. splendens* (Sp), *S. baicalensis* (Sb), *S. indicum* (Si) and *A. thaliana* (At). Bootstrap support values (percentages) from 1000 replicates are shown next to relevant clades. **b** Syntenic analysis of Danshen tanshinone biosynthetic gene cluster by comparison to *S. splendens*, *S. baicalensis* and *S. indicum*. **c** Syntenic analysis of the Danshen CYP71D subfamily gene cluster by comparison to *S. splendens* and *S. baicalensis*. Source data underlying Fig. 8b and c are provided as a Source Data file.

In the collinear region corresponding to the Danshen tanshinone biosynthetic gene cluster, there were three diterpene cyclases/synthases in *S. indicum* and *S. splendens* (albeit these are scattered across two scaffolds in the latter), and seven diterpene cyclases/ synthases in *S. barcalensis* (Fig. 8a, b). There are orthologs of *SmCPS1* and *SmCPS2* in the isogenic regions of *S. barcalensis* (*SbTPS3* (Sb06t19660) and *SbTPS5* (Sb06t19680)), and *S. splendens* (*SsTPS2* (Saspl_048790.T1) and *SsTPS3* (Saspl_017770.T1)). But there is only one ortholog *SiTPS1* (rna17299) in *S. indicum* (Fig. 8b). Notably, *SmKSL1* is phylogenetically distinguished by a relatively recent relictual domain loss event^31^. This is still evident in the orthologs from *S. barcalensis* (*SbTPS2*) and *S. splendens* (*SsTPS1*) that is found on the same scaffold as the *SmCPS1* ortholog *SsTPS2*. However, the ortholog in Sesame (*SiTPS2*) exhibits the more ancestral three-domain structure, which suggests that the domain loss event may have occurred prior to the divergence of the *Salvia* and *Scutellaria* genera relative to the *S. indicum* lineage. Intriguingly, although *TPS/CYPs* gene pair are the core components of terpenoid biosynthetic gene clusters^32^, only Danshen and *S. splendens* have *CYPs* in this region (Fig. 8b). Though *S. splendens* has two CYP76AH subfamily members, *SsCYP76AH1.1* (Saspl_017771.T1) and *SsCYP76AH1.2* (Saspl_017768.T1), full-length cDNAs are not evident for these two genes, suggesting that these may be inactive. There is an apparent ortholog of *SsCYP76AH1.1* between *SmCPS1* and *SmKSL1* in the Danshen tanshinone biosynthetic gene cluster, but a premature termination codon indicates that this *SmCYP76AH1.1* is also inactive. In contrast, *S. indicum* and *S. barcalensis* have no CYPs in the corresponding region. Notably, the pair of class II diterpene cyclases in this region of the Danshen and *S. splendens* genomes may have originated from *SiTPS1* by tandem duplication, while *CYP76AH1* and *CYP76AH3* seem to have emerged following divergence of the Danshen and *S. splendens* lineages.

The Danshen CYP71D subfamily gene cluster did not have orthologs in *S. indicum* and *S. barcalensis* (Supplementary Fig. 14). In the case of *S. barcalensis*, while nine genes on pseudochromosome 1 are orthologous to the four upstream and five downstream genes from Danshen, no CYP71D subfamily members are present in this region (Fig. 8c). Three collinear blocks can be found in *S. splendens* (Fig. 8c), each of which contains a CYP71D subfamily member. These are saspl_041816.T1, saspl_008326.T1, and saspl_029000.T1, which seem to be related to the Danshen CYP71D clade targeted here, although full-length cDNAs are not evident for any of these putative *S. splendens* CYP71D subfamily members. Among them, saspl_041816.T1 and saspl_029000.T1 have the highest homology with *CYP71D375*, while saspl_008326.T1 has the highest homology with *CYP71D411*. Thus, this CYP71D clade, which seems to be responsible for heterocyclization to form the D-ring, seems to have emerged in the *Salvia* genus.

## Discussion

Generation of the 14,16-epoxy D-ring not only distinguishes the tanshinones within the phenolic abietane-type *nor*-diterpenoids found throughout the Lamiaceae family, but also provides pharmaceutical import as the target for sulfonation to generate a clinically relevant derivative. While tanshinone biosynthesis has been investigated for many years^10–13^, the origin of this heterocycle has remained unknown. Here, we improved upon previously reported draft genomes by sequencing a highly homozygous line of Danshen. This highlighted the expansion of a clade within the CYP71D subfamily, with the expression of four of these found to be tightly correlated with tanshinone biosynthesis. Indeed, our results indicate that at least two of these, CYP71D373 and CYP71D375, play important roles in forming the characteristic 14,16-epoxy D-ring. This is supported by both the observed relative increase in intermediates that do not contain the heterocyclic D-ring in *CYP71Ds*-RNAi plants and recombinant biochemical activity. The promiscuity observed with CYP71D375 further suggests that tanshinone biosynthesis might operate as a metabolic grid, with heterocyclization to form the D-ring occurring after loss of C20 (Fig. 6a). While CYP71D373 and CYP71D375 exhibit partial functional redundancy, the substantial sequence divergence between these (aa sequence identity of ~70%) suggests the presence of selective pressure for the retention of both. Given their differential induction^10^, this may reflect distinct roles in inducible versus constitutive production of tanshinones for *CYP71D373* and *CYP71D375*, respectively.

In contrast, CYP71D411 seems to act as earlier acting C20 hydroxylase. However, this activity overlaps that previously reported for CYP76AK1, which is supported by metabolite accumulation upon RNAi knockdown and biochemical activity^9^. Moreover, a number of CYP76AK1 orthologs have been identified in other Lamiaceae plant species that also produce C20 oxygenated derivatives of phenolic abietane-type diterpenoids^33,34^, further supporting this functional assignment. While we speculate that the appearance of CYP71D411 was driven by a need for increased flux towards tanshinone biosynthesis, consistent with the notable effect of these pigmented (reddish) diterpenoids on the coloration of the Danshen root periderm^22,35^, this is purely hypothetical. Regardless, this distinct biochemical activity helped direct mutational analysis of the enzymatic structure-function relationships underlying the heterocyclization activity exhibited by CYP71D375. The results indicate an important role for substrate positioning within the active site, with particularly important roles played by A301 and, especially, L366.

Perhaps more interestingly, the improved genome sequence reported here provides insight into the evolution of both the phenolic abietane-type diterpenoids and tanshinones. It has previously been shown that other Lamiaceae plant species use orthologs of the genes firstly discovered in Danshen to produce 11-hydroxyferruginol—i.e., *SmCPS1, SmKSL1, CYP76AH1*, and *CYP76AH3*^33,34,36–38^. Notably, these are exactly the genes found in the larger tanshinone biosynthetic gene cluster defined here. Given that collinear regions can be found in *S. splendens*, this might be more accurately termed a ferruginol biosynthetic gene cluster. It seems to have evolved in the lineage that gave rise to the *Salvia* genus, much as recently reported for triterpenoid biosynthetic gene cluster in the *Arabidopsis* genus^39^. Such co-clustering of *CPS*, *KSL*, and a *CYP76AH* subfamily member containing all the genes necessary for the production of ferruginol is consistent with the widespread production of phenolic abietane-type diterpenoids in the *Salvia* genus. More specific to tanshinone production is elucidation of the biosynthetic origins of the characteristic D-ring heterocycle reported here, as the relevant CYP71D clade I seems to have been expanded upon, in large part as a tandem gene array, in Danshen. While CYP71D subfamily members are present in the collinear regions of *S. splendens*, given that this species is not thought to produce such heterocyclic D-ring containing abietane diterpenoids^40^, as well as the highly divergent activities observed in this subfamily^17^, we hypothesize that it was neofunctionalization of this clade that led to the observed characteristic 14,16-epoxidation activity in Danshen. In addition to clade I, the CYP71D clade III also seems to have been expanded in the Danshen genome, and may play a role in biosynthesis of other characteristic metabolites such as salvianolic acid B^40^.

In conclusion, we sequenced a highly homozygous line of Danshen and substantially improved the genome assembly comparing to the existing ones^18,19^. The improved genome assembly enabled the discovery of a large biosynthetic gene cluster associated with the early steps in tanshinone biosynthesis. More specifically, this biosynthetic gene cluster enables the production of at least ferruginol, and may be more widespread in *Salvia*, consistent with the broad appearance of such phenolic abietane-type diterpenoids in this genus. By contrast, elucidation of the biosynthetic origins of the D-ring reported here provides insight into the evolution of this characteristic heterocycle. In particular, this seems to have arisen from the expansion of a clade within the CYP71D subfamily that underwent neofunctionalization to catalyze the formation of this 14,16-epoxide. Accordingly, our results provide insight into not only the more specific biosynthetic origins and evolutionary derivation of the medically relevant tanshinones, but also that of the more general phenolic abietane-type diterpenoids, which are more broadly distributed in the Lamiaceae family.

## Methods

### Plant materials and chemicals

In order to reduce heterozygosity, line bh2-7, which has been subjected to six cycles of self-pollination, was used in this study. Seedlings of this line were grown in sterile culture, with the shoot tips used for further propagation without hormones. The resulting plant material was used for DNA sequencing and Hi-C library construction. The transformed seedlings were transplanted in a soil:vermiculite (3:1) system and grown in a greenhouse under the same temperature and light regime^22^. For RNA sampling, self-pollinated progeny of bh2-7 was subjected to low-temperature vernalization outside (February to March), and then moved into greenhouse for flowering. The *CYP71Ds*-RNAi and wild-type plants were grown in the greenhouse for three months before analysis. Tanshinone I, tanshinone IIA, **1**, **2** and sugiol (**3**) were purchased from Chengdu Must Bio-Technology Co., Ltd (Sichuan, China). Miltirone (**4**), neocryptotanshinone (**8**) and methylenedihydrotanshinquinone (**11**) were purchased from Beijing Rongchengxinde Co., Ltd (Beijing, China). 2-isopropyl-8-methylphenanthrene-3,4-dione (here after Ro, **5**), 4-methylenemiltirone (**6**) and 16-hydroxyRo (**9**) were kindly provided by Prof. Jungui Dai, Prof. Wude Yang, and Prof. Kun Gao, respectively. The purity of these standards was > 95%.

### Genome sequencing

Genomic DNA was extracted from leaves with a standard CTAB method. DNA purity was verified by spectroscopic analysis with NanoDrop Spectrophotometers (Thermo Scientific). The Illumina paired-end genome library was constructed according to the standard protocol and seven paired-end Illumina WGS libraries were constructed with multiple insert sizes (200 bp, 450 bp 500 bp, 800 bp, 2 kb, 5 kb, 10 kb, and 20 kb), which were then sequenced on a HiSeq 2500 platform.

Library construction for PacBio sequencing was carried out using the protocols recommended by the manufacturer. A 20 kb single-molecule read library was constructed, which was then sequenced with a PacBio RSII Sequencer (Pacific Biosciences, USA) using the P6-C4 chemistry system.

The Hi-C library was prepared following standard procedures by Annoroad Genomics (Beijing, China) following their standard procedure^41^. The sequencing reads were mapped to the draft genome assembly by BWAmem. Then the contigs were clustered onto super-scaffolds with LACHESIS (http://shendurelab.github.io/LACHESIS).

### Estimation of genome size

The genome size was measured by flow cytometry according to the protocol described by Dolezel et al.^42^. Briefly, young seedlings were chopped up with a sharp razor blade for about 1 minute in LB01 buffer (15 mM Tris, 2 mM Na_2_EDTA, 0.5 mM spermine tetrahydrochloride, 80 mM KCl, 20 mM NaCl, 0.1% (v/v) Triton X-100, *β*-mercaptoethanol to 15 mM; pH 7.5). The homogenate was mixed by pipetting up and down several times, then filtered through a 42-μm nylon mesh into a labeled sample tube. Plant cell nuclei were stained by adding DNA propidium iodide and RNase A at a concentrate of 50 μg mL^−1^. The mixture was gently shaken and incubated on ice before analysis, with occasional shaking to keep in suspension. Two sequenced species, tomato and *Cusumis sativus*, were used to analyze the genome size of Danshen. The genome size was further evaluated by k-mer frequency analysis, based on Illumina short reads using the k-mer Analysis Toolkit (http://www.earlham.ac.uk/kat-tools).

### Genome assembly and annotation

Two intermediate assembly versions of the genome were separately generated by DISCOVAR, using the Illumina reads (v0.1), and Falcon (v1.7.4), using the PacBio reads (v0.2). These were then merged using the HABOT (hybrid assembly of third-generation sequencing 2; https://github.com/asarum/HABOT2) software (1gene Corp., Hangzhou, China)^43^. A final round of scaffolding and gap filling was performed using Illumina reads to obtain a v1.0 of the Danshen genome. A more detailed protocol of genome assembly methods can be found in Supplementary Method 1.

The gene prediction pipeline used here combined ab initio gene prediction, homologous sequence searching and transcriptome sequence assembly. A detailed description for the prediction of genes, repeat sequences, non-coding RNA and tRNA can be found in Supplementary Method 2.

### Genome evolution

Danshen gene evolution was analyzed by identifying orthologous genes from selected species – i.e., *A. thaliana*, *C. annuum*, *S. lycopersicum*, *S. tuberosum*, *B. hygrometrica*, *M. guttatus*, *A. paniculata*, *S. indicum*, *U. gibba, S. splendens*, and *S. barcalensis*. Proteins from all selected species were analyzed via all-by-all blastp. Similar gene pairs were then clustered into groups using OrhtoMCL (v2.0.2)^44^. The single copy orthologous genes were used to construct the phylogenetic tree using the maximum likelihood method in the PhyML (v3.0)^45^ software package. More detailed description of gene family and genome evolution analyses can be found in Supplementary Method 3.

The dynamic evolution of gene families was investigated using CAFE software (v2.1,–filter) with a probabilistic graphical model^20^. Finally, gene families with significantly different sizes (P ≤ 0.05) in each species were annotated.

We performed syntenic searches to compare the specific regions containing diTPSs and CYP71Ds from Danshen with the most closely related species, particularly *S. splendens, S. barcalensis* and *S. indicum*. Syntenic blocks were assigned via all-by-all BLASP with cutoffs of identity ≥ 40% and *e*-value ≤ 1e^−10^. Synteny comparison was performed using JCVI with LASTAL as sequence alignment tool with default parameters. Microsynteny visualization was drawn using a modified version of JCVI^46^.

### Transcriptome analysis and cDNA cloning

For transcriptome analysis, five tissues at the flowering stage (root, stem, leaf, calyx, and petal), together with roots of *CYP71Ds*-RNAi and paired WT, were collected, with three biological replicates for each. Total RNA was extracted using a quick RNA isolation kit (HuaYueYang Biotechology, Beijing, China) according to the manufacturer’s instructions. Then the RNA is shipped to the Novogene company (www.novogene.com) for quality estimation, library construction and sequencing. The RNA quality was determined using an Agilent 2100 Bioanalyzer. The cDNA libraries were sequenced on one lane for 151 cycles from each end of the cDNA fragments on a HiSeq 2500 (Illumina)^47^. The full-length cDNA for *CYP71D373*, *CYP71D375*, *CYP71D411*, and *CYP71D464* were identified based on the genome and transcriptome sequencing data. The open reading frames were further cloned into the pESC-His vector for functional analysis.

### Plant transformation for RNAi of CYP71D candidates

The region comprising nucleotides 823-1276 from *CYP71D411* was cloned and transferred into the pK7GWIWG (II) binary vector using Gateway technology. The resulting pK7GWIWG-CYP71D was introduced into *Agrobacterium tumefaciens* strain EHA105 by electroporation. Cells were cultured to an OD600 of 0.6, and then collected by centrifugation. The cells were resuspended in liquid Murashige and Skoog (MS) medium for genetic transformation. Before transformation, leaves or petioles were cut into disks and precultured for 2 days on MS basal medium supplemented with 2.0 mg L^–1^ 6-benzyladenine. The prepared disks were incubated with cell suspension by shaking for 15 min, and then cocultured on MS medium for 2 days. The leaf disks were selected on MS medium supplemented with 2.0 mg L^–1^ 6-benzyladenine, 50 mg L^–1^ kanamycin, and 225 mg L^–1^ timentin. After 2-3 rounds of selection (10 days each), the regenerated buds with GFP fluorescence were transferred to MS medium supplemented with 25 mg L^–1^ kanamycin for root formation and elongation. Rooted plantlets were cultured on MS medium for about 1 month, then transplanted to soil and vermiculite (3:1) and covered by beakers to maintain humidity for 1 week.

### Metabolomics profiling using LC-qTOF-MS and GC-QqQ-MS

Two independent analytical platforms were employed to acquire and analyze the metabolomic data^22^. Briefly, LC-qTOF-MS analysis of methanol extracts was used for global unbiased metabolite detection. GC-QqQ-MS analysis of hexane extracts was optimized for detection of miltiradiene, abietatriene and ferruginol. LC-qTOF-MS analyses was carried out using an Agilent 1290 Infinity UPLC system with a VWD detector at 285 nm. An Agilent ZORBAX RRHD SB-C18 column (2.1 × 100 mm, 1.8 μm) was used for chromatographic separation. Mass spectrometry was acquired with an Agilent 6540 qTOF equipped with an electrospray ionization (ESI) source operating in positive ion mode. For full-scan MS analysis, the data acquisition range of mass-to-charge ratio (*m/z*) was from 100 to 1000. The nebulization gas was set to 40 pounds per square inch. The flow rate of drying gas and sheath gas was set at 10 L min^–1^ and 11 L min^–1^ at 350 °C, respectively. The capillary voltage was set to 4000 V and the acquisition rate was set at 0.5 s. GC-QqQ-MS analyses were performed on an Agilent 7890 A GC system with a 7000B triple quadruple MS detector at electron impact ionization. The column was an Agilent DB-5ms (30-m × 0.25-mm i.d., 0.25-μm film thickness; Agilent J&W Scientific). Helium was used as the carrier gas for GC with a flow rate of 1.0 mL min^–1^. The injector and transfer line temperature was 280 °C. The following temperature program was used: 50 °C for 2 min, then a linear ramp at a rate of 20 °C min^–1^ to 200 °C followed by a 5 °C min^–1^ linear ramp to 300 °C, and held at 300 °C for 10 min.

### Heterologous expression in yeast and in vitro activity assay

The epitope-tagged pESC-His vectors carrying *CYP71D373*, *CYP71D375*, *CYP71D411,* or *CYP71D464* were each transformed into the yeast strain WAT11, which enables catalytic activity of plant CYPs by also expressing the *A. thaliana* NADPH-CYP reductase ATR1^23^. WAT11 transformed with empty pESC-His was employed as control. TE buffer was prepared with 50 mM Tris-HCl, 1 mM EDTA, pH 7.5. The cells were recovered by centrifugation at 5000 g for 4 min, resuspended in TEK (0.1 M KCl in TE) to a concentration of 0.5 g wet cells per mL and left at room temperature for 5 min. The cells were again recovered by centrifugation and resuspended in TESB (0.6 M sorbitol in TE). Cells were broken up at 2-6°C by a cryogenic homogenizer. After centrifugation at 20000 g for 20 min, microsomes were precipitated by adding NaCl to the supernatant to a final concentration of 0.15 M and polyethylene glycol PEG4000 to a final concentration of 0.1 g mL^−1^. Pellets were resuspended in TEG (20% (v/v) glycerol in TE)^13^. In vitro activity assays were performed in a 500 μL reaction system that included 100 mM Tris-HCl (pH 7.5) and 500 μM NADPH, along with a regenerating system (5 mM glucose-6-phosphate, 1 unit glucose-6-phosphate dehydrogenase, 5 μM FAD, and 5 μM FMN), 0.5 mg microsomal protein, and 100 μM of the substrate. The reactions were incubated at 30 °C for 4 hours with shaking, and then extracted with 500 μL of ethyl acetate.

### Isolation of products and NMR analysis

Dried Danshen root was ground up and the resulting powder (200 g) was soaked in 2 L ethyl acetate overnight, then the mixture homogenized by sonication for 30 minutes. The organic phase was separated, dried, and the residue dissolved in 20 mL acetonitrile for isolation of 16-hydroxymiltirone (**7**). For isolation of sufficient amounts of 20-hydroxysugiol (**12**) for NMR analysis, the in vitro enzymatic reaction system was expanded from 500 μL to 100 mL. The assay was incubated in a shaker at 100 rpm min^−1^ for six hours. Ethyl acetate (100 mL) was then added to the assay, followed by sonication for 20 minutes. After centrifugation at 4000 g for ten minutes, the ethyl acetate layer in the supernatant was collected and dried under nitrogen, then the residue dissolved in 5 mL acetonitrile. Compounds **7** and **12** were purified using a Shimadzu LC-20AR preparative liquid chromatography system, with a J’sphere ODS-M80 column (20 × 250 mm, 4 μm). The mobile phase for purification of **7** was a 3.5:6.5 mixture of water and acetonitrile (v/v), while a 3:7 mix of water and acetonitrile (v/v) was used as mobile phase for purification of **12**, with a flow rate of 8 mL min^−1^ in each case. For chemical structure characterization, ^1^H NMR (600 MHz), ^13^C NMR (100 MHz), and two-dimensional (2D) NMR spectra were recorded with a Bruker DRX Avance-600 (Bruker Co., Switzerland) NMR spectrometer. The observed chemical shift values are reported in ppm.

### Modeling docking and mutagenesis

CYP71D373, CYP71D375, CYP71D411, and CYP71D464 were modeled using SwissModel, with the structure of the most closely related CYP76AH1 (5YLW) serving as the template^24^. The coordinates of the heme protoporphyrin were then inserted in the modeled CYP71D structures from that found in 5YLW. Miltirone was protonated and docked into the structures with AutoDock Vina^48^. Molecular distances were calculated using PyMol^49^ (http://www.pymol.org). Substitutions for the selected residues in CYP71D375 were constructed by PCR using the primers listed in Supplementary Table 14.

### Reporting summary

Further information on research design is available in the Nature Research Reporting Summary linked to this article.

## Supplementary information

Supplementary Information

Peer Review File

Reporting Summary

Description of Additional Supplementary Files

Supplementary Data 1-4

## Data Availability

The data supporting the findings of this work are available within the paper and the Supplementary Information files. A reporting summary for this article is available as a Supplementary Information file. The data sets generated and analyzed during this study are available from the corresponding author upon request. The genome sequence and assembly are available at NCBI BioProject PRJNA682867. The databases of KEGG (http://www.genome.jp/kegg/), Swissprot and TrEMBL (http://www.uniprot.org/), and InterPro (https://www.ebi.ac.uk/interpro/) are used for data analyses in this study. Source data are provided with this paper.

## Source data

Source Data
