# Supplementary material for: Expansion within the CYP71D subfamily drives the heterocyclization of tanshinones synthesis in Salvia miltiorrhiza
Source: Nat Commun. 2021 Jan 29;12:685. doi: 10.1038/s41467-021-20959-1 (PMC7846762; doi:10.1038/s41467-021-20959-1)
Supplement: Supplementary file 1 — Supplementary Information [file 41467_2021_20959_MOESM1_ESM.docx]

**Supplementary Information**

**Expansion within the CYP71D subfamily drives the heterocyclization of tanshinones synthesis in *Salvia miltiorrhiza***

**Supplementary Figures**

**
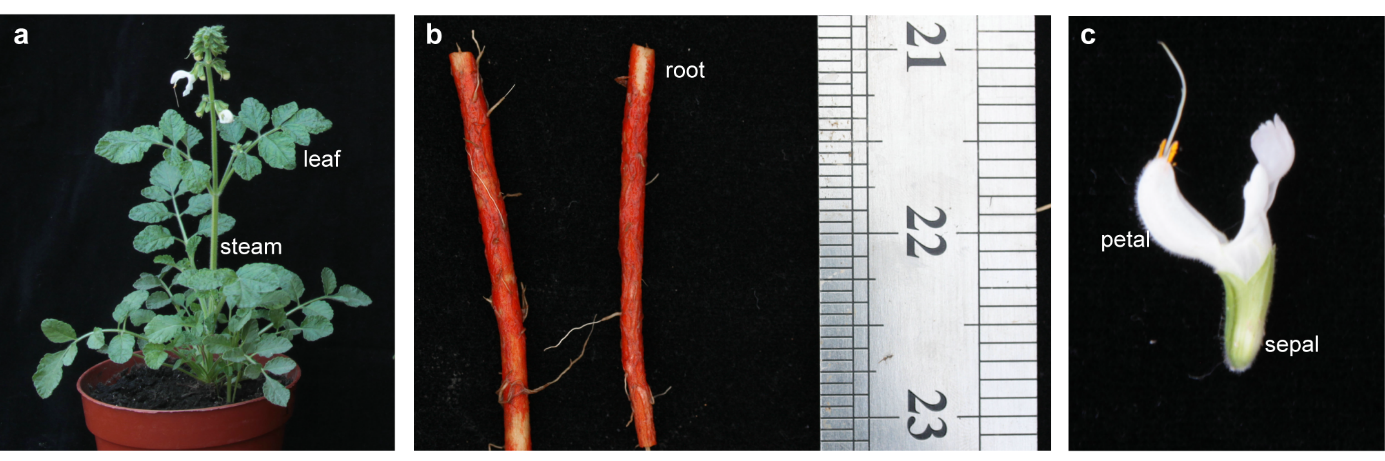
**

**Supplementary Fig. 1 Plant material used in this study.**

(**a**) *Salvia miltiorrhiza* var. alba line bh2-7 plant. (**b**) The root of line bh2-7. (**c**) The flower of *Salvia miltiorrhiza* var. alba line bh2-7.

**
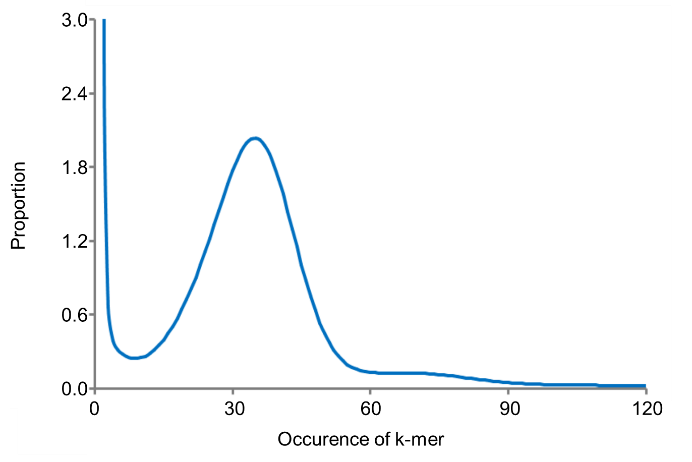
**

**Supplementary Fig. 2 k-mer analysis to estimate the sesame genome size.**

The figure shows frequency of 17-mer which are 17 bp tilling sequences from the reads (after filtering) of short-insert size libraries. We identified 22,448,863,609 of 17-mers from 26.73 Gb data. The genome size can be estimated by (total k-mer number) / (the volume peak), which was thus estimated as 623.58 Mb.

**
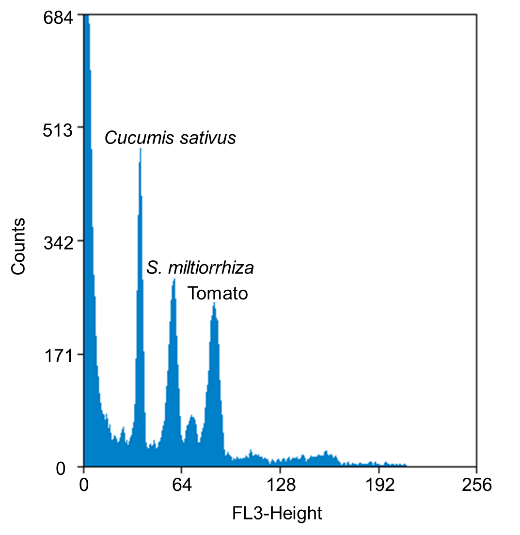
**

**Supplementary Fig. 3 Flow cytometric analysis of the genome size of Danshen.** *Cucumis sativus* and tomato was used as internal biological reference. The genome size was estimated to be 622 Mb.

**
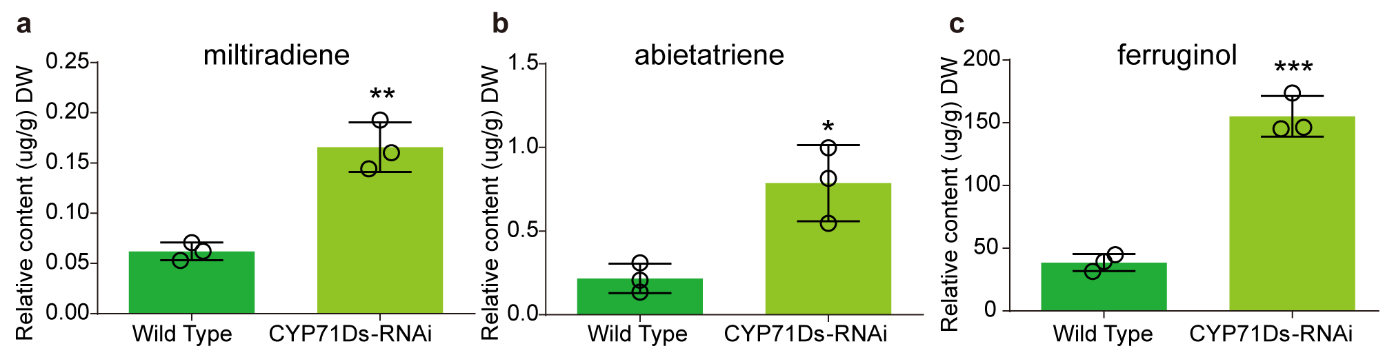
**

**Supplementary Fig. 4. Content of miltiradiene (a), abietatriene (b), and ferruginol (c) in the *CYP71Ds*-RNAi lines as compared with WT analyzed using GC-MS.** The relative concentration obtained by comparison with an internal standard tetracosan, error bars represent standard deviations (SD) (n =3 biologically independent samples; * P < 0.05, **P < 0.01, ***P < 0.001 by 2-sided Student’s t test). Source data are provided in Source Data file.

**
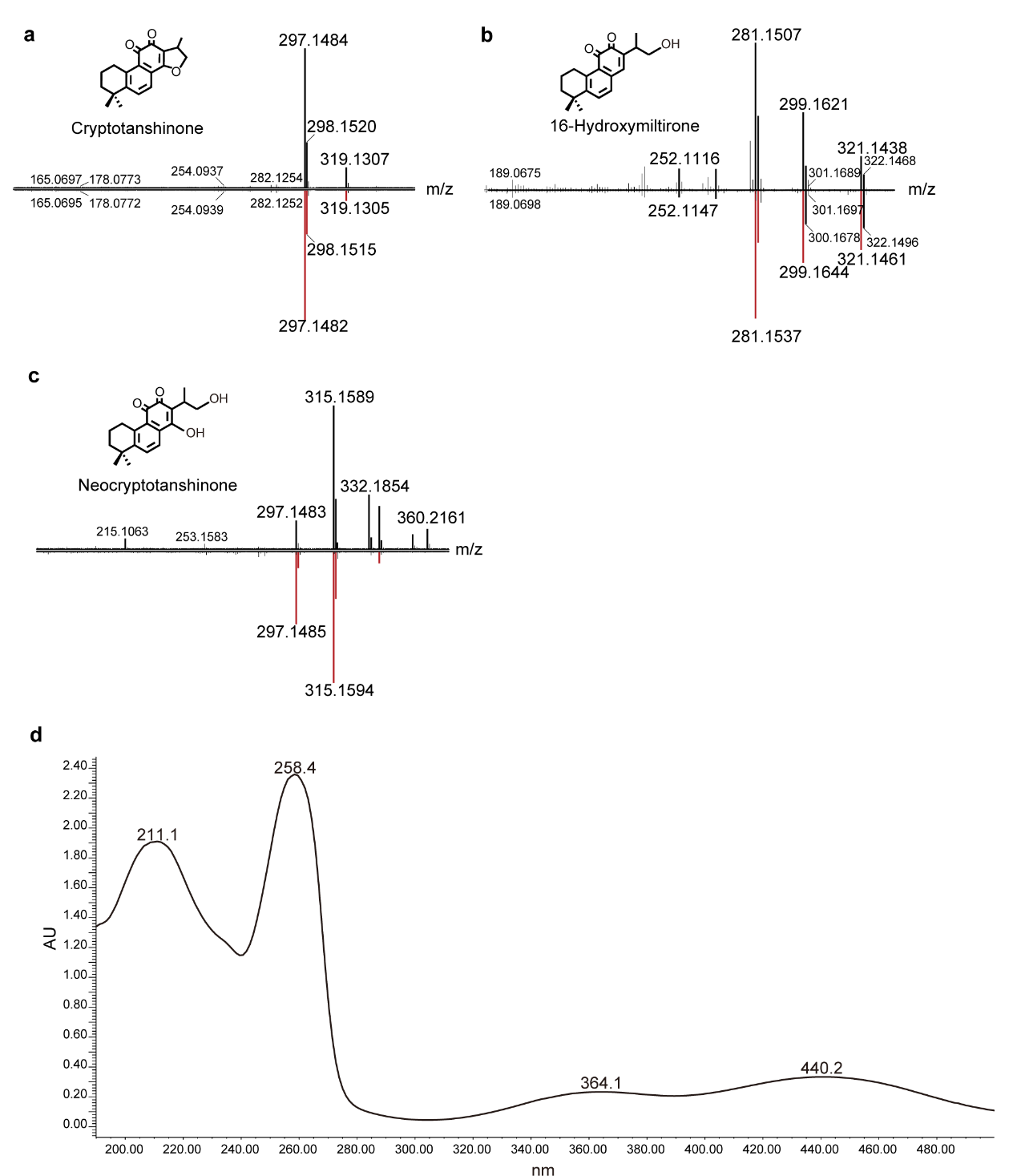
**

**Supplementary Fig. 5 Identification of the CYP71D375 products from miltirone.**

Butterfly plots of mass spectra for, (**a**) product **1** and authentic standard for cryptotanshinone, both with retention time (RT) = 3.75 min; (**b**) product **7** and authentic standard for 16-hydroxymiltirone, both with RT = 2.50 min; (**c**) Product **8** and authentic standard for neocryptotanshinone, both with RT = 3.10 min. Enzyme reaction products are colored black and the standards are colored red in each case.

**a**


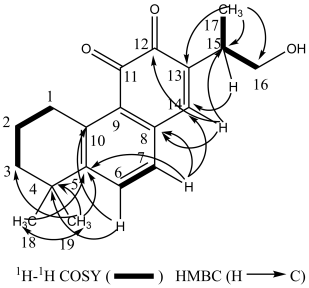


**b**


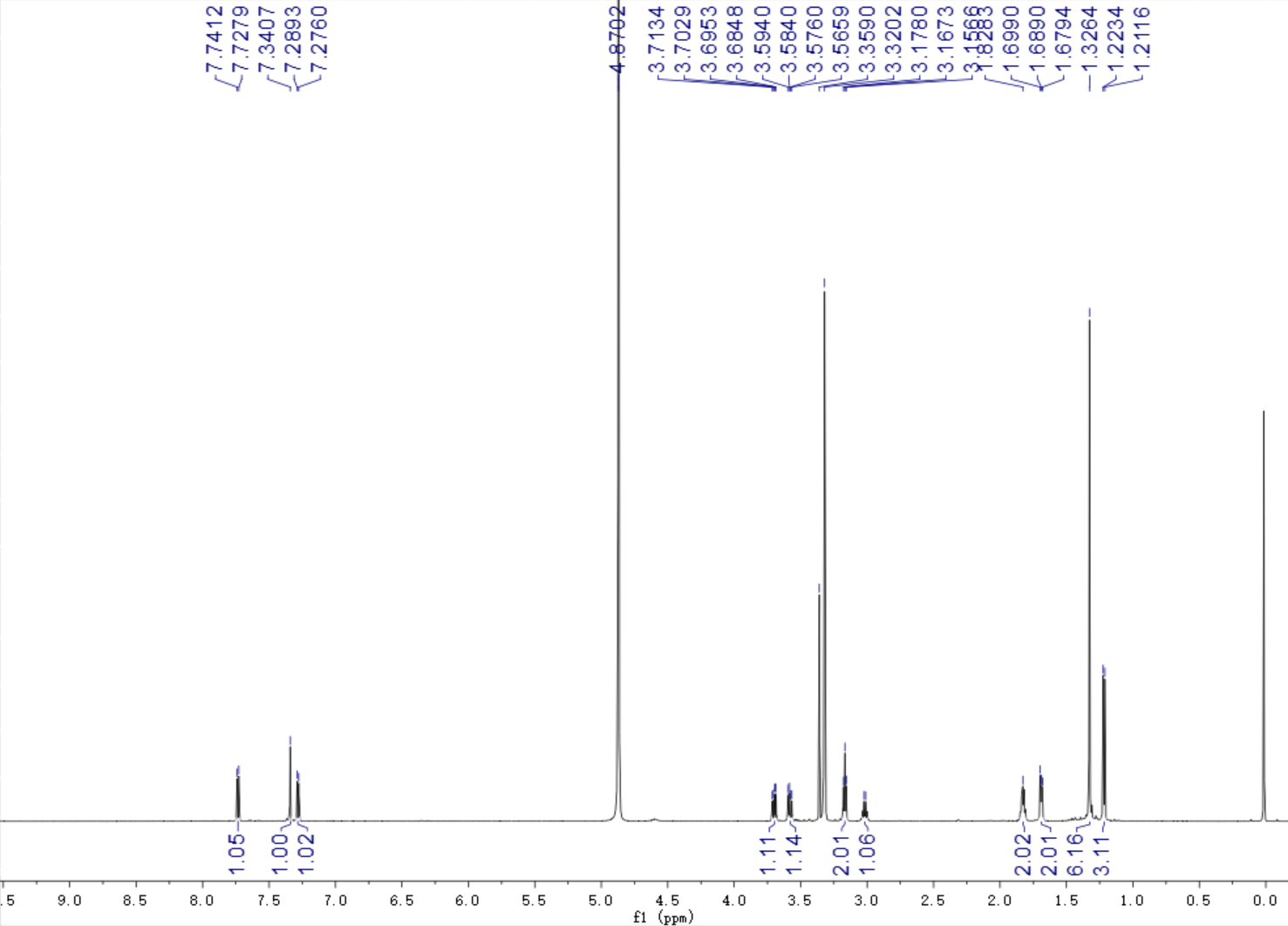


**c**


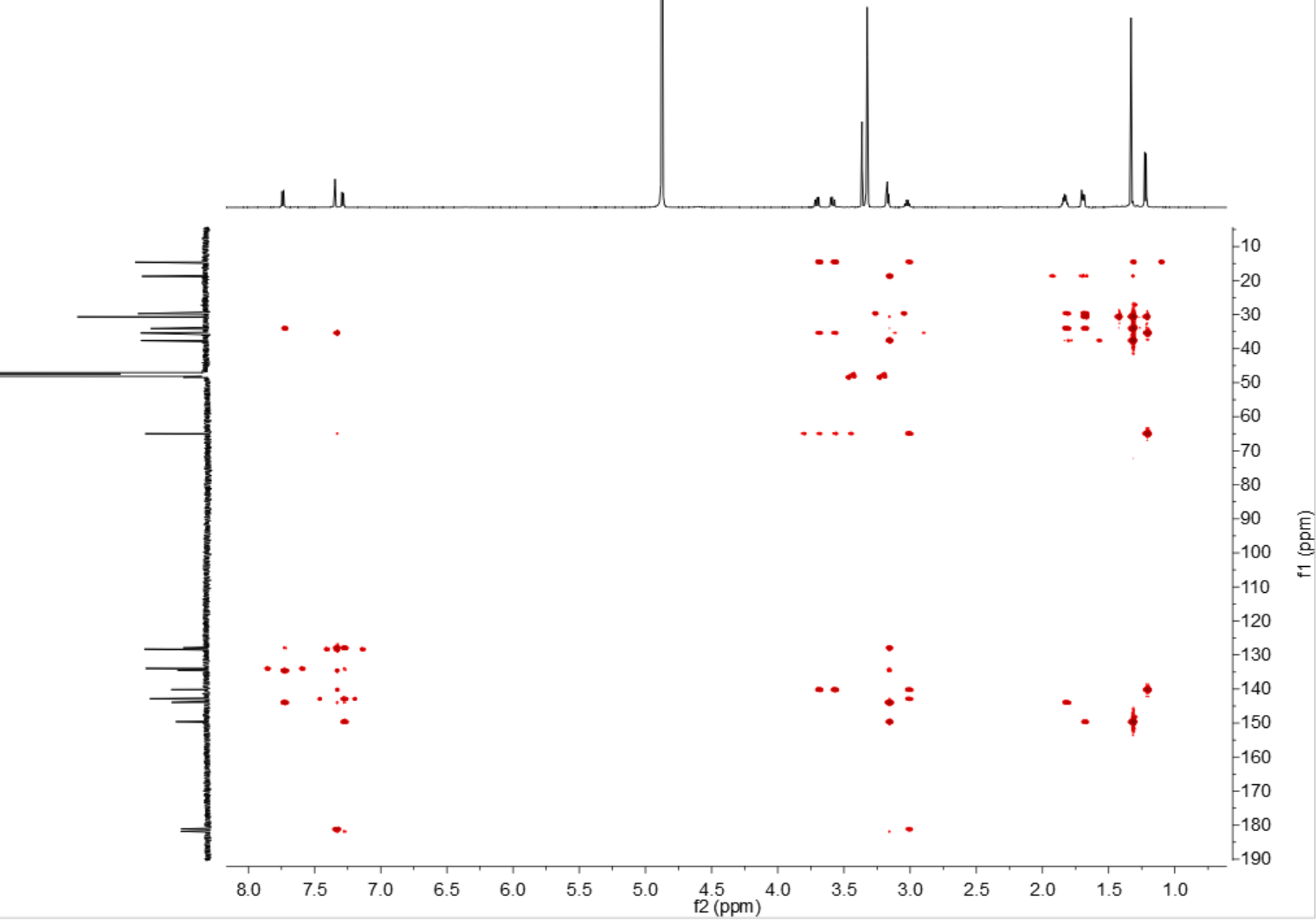


**d**


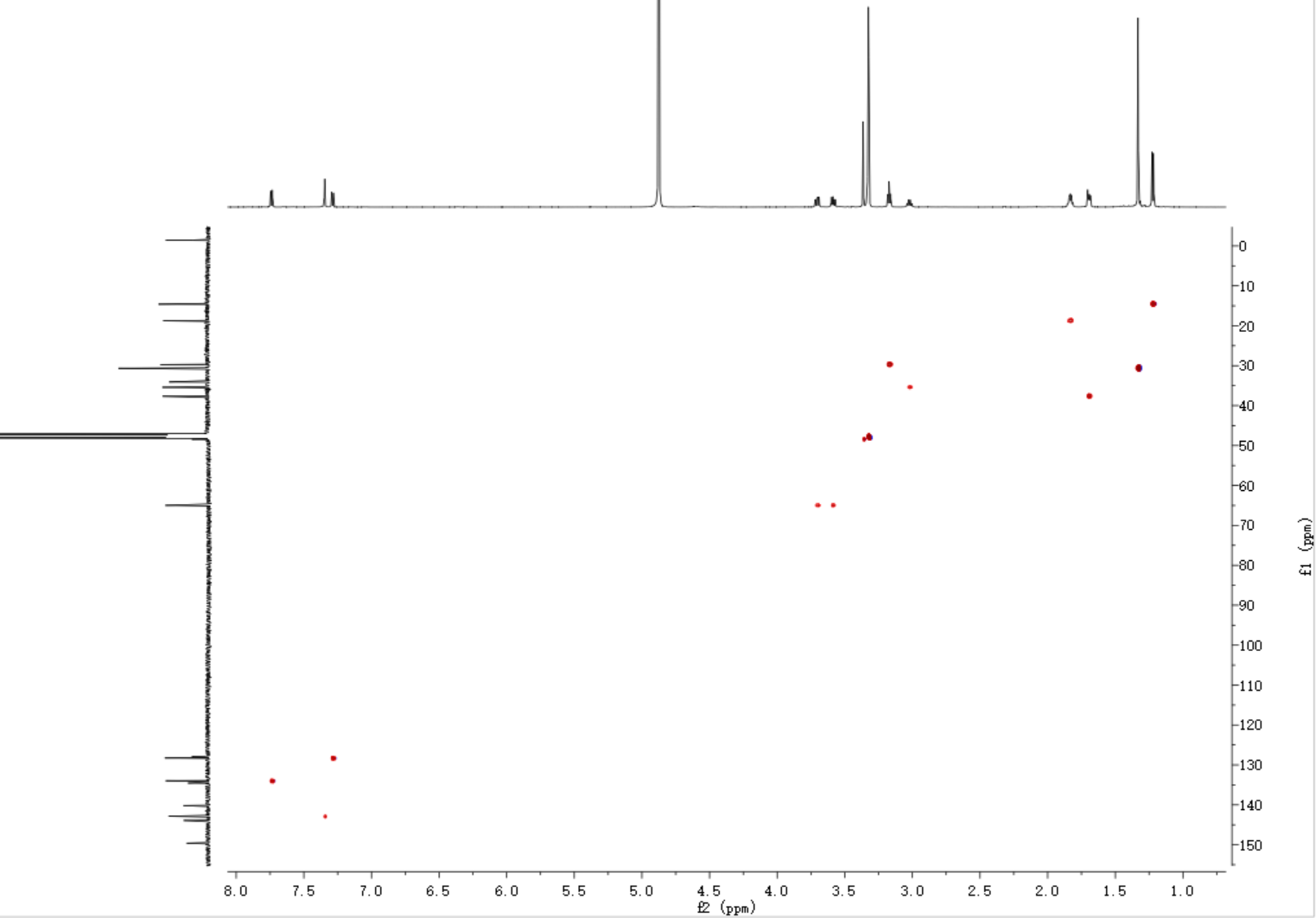


**e**


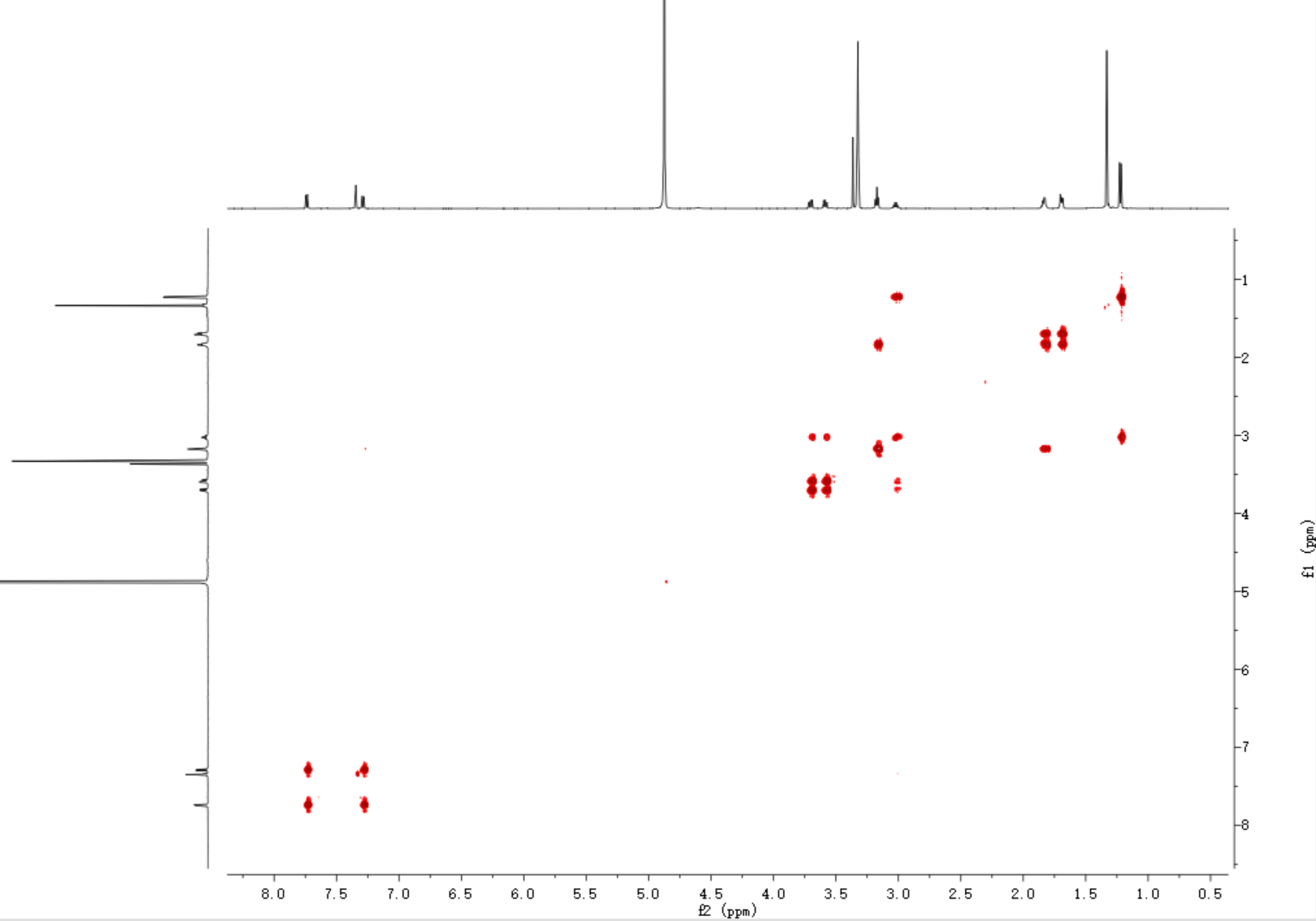


**f**


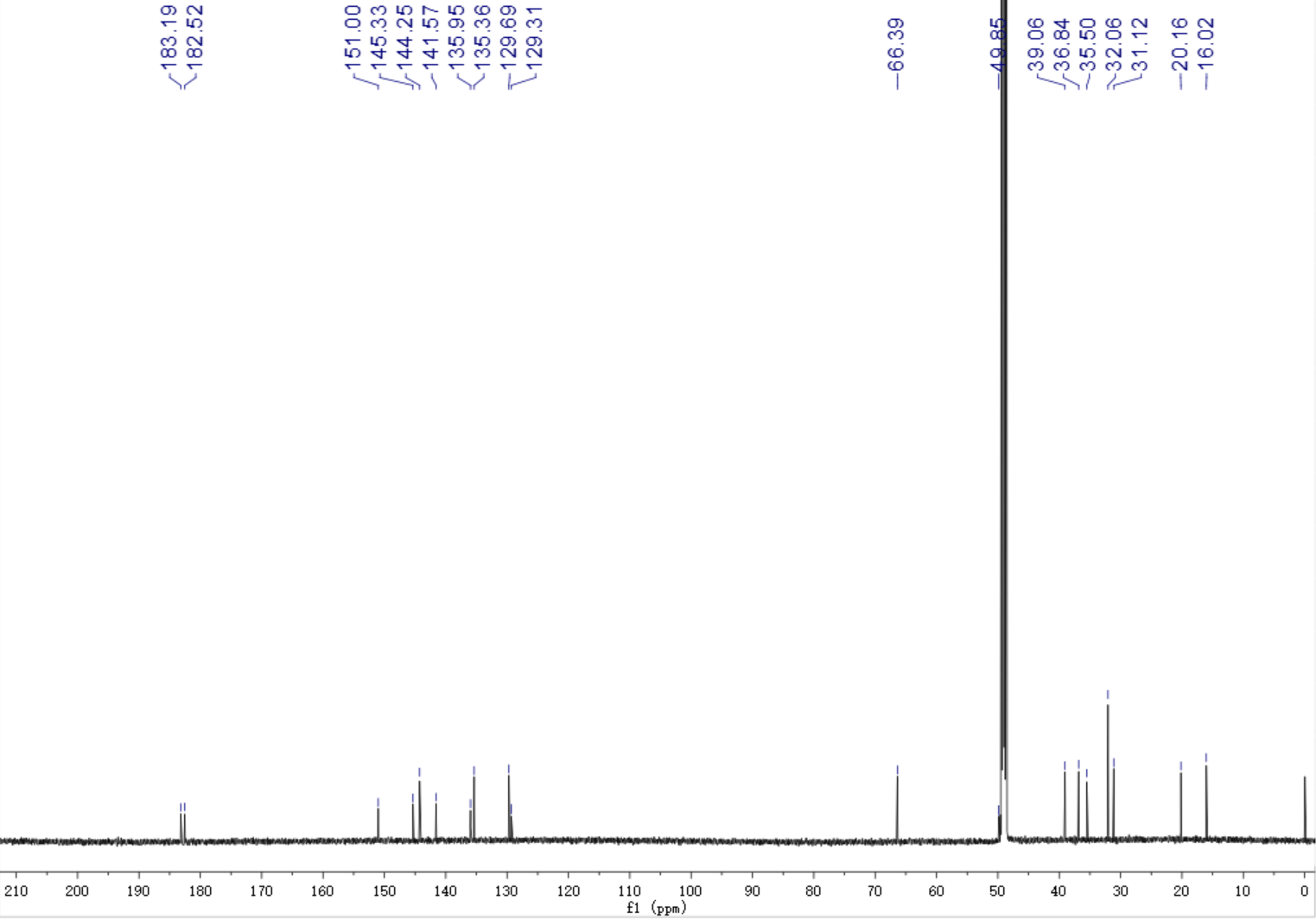


**Supplementary Fig. 6 Structural characterization of 16-hydroxymiltirone (7) by NMR.**

Chemical shift assignments, based on (**a**) assigned HMBC and COSY correlations, (**b**) ^1^H NMR spectrum in CH_3_DO, (**c**) HMBC spectrum in CH_3_DO, (**d**) HSQC spectrum in CH_3_DO, (**e**) HH in CH_3_DO COSY spectrum, (**f**) ^13^C NMR spectrum in CH_3_DO.

^1^H NMR data (600 MHz, MeOD) δ 7.73 (d, *J* = 8.0 Hz, 1H), 7.34 (s, 1H), 7.28 (d, *J* = 8.0 Hz, 1H), 3.70 (dd, *J* = 10.8, 6.3 Hz, 1H), 3.58 (dd, *J* = 10.8, 6.0 Hz, 1H), 3.17 (t, *J* = 6.4 Hz, 2H), 3.02 (dd, *J* = 13.3, 6.8 Hz, 1H), 1.83 (m, 2H), 1.69 (m, 2H), 1.33 (s, 6H), 1.22 (d, *J* = 7.1 Hz, 3H); ^13^C NMR data (151 MHz, MeOD) δ 183.19, 182.52, 151.00, 145.33, 144.25, 141.57, 135.95, 135.36, 129.69, 129.31, 66.39, 49.85, 39.06, 36.84, 35.50, 32.06, 31.12, 20.16, 16.02.

**
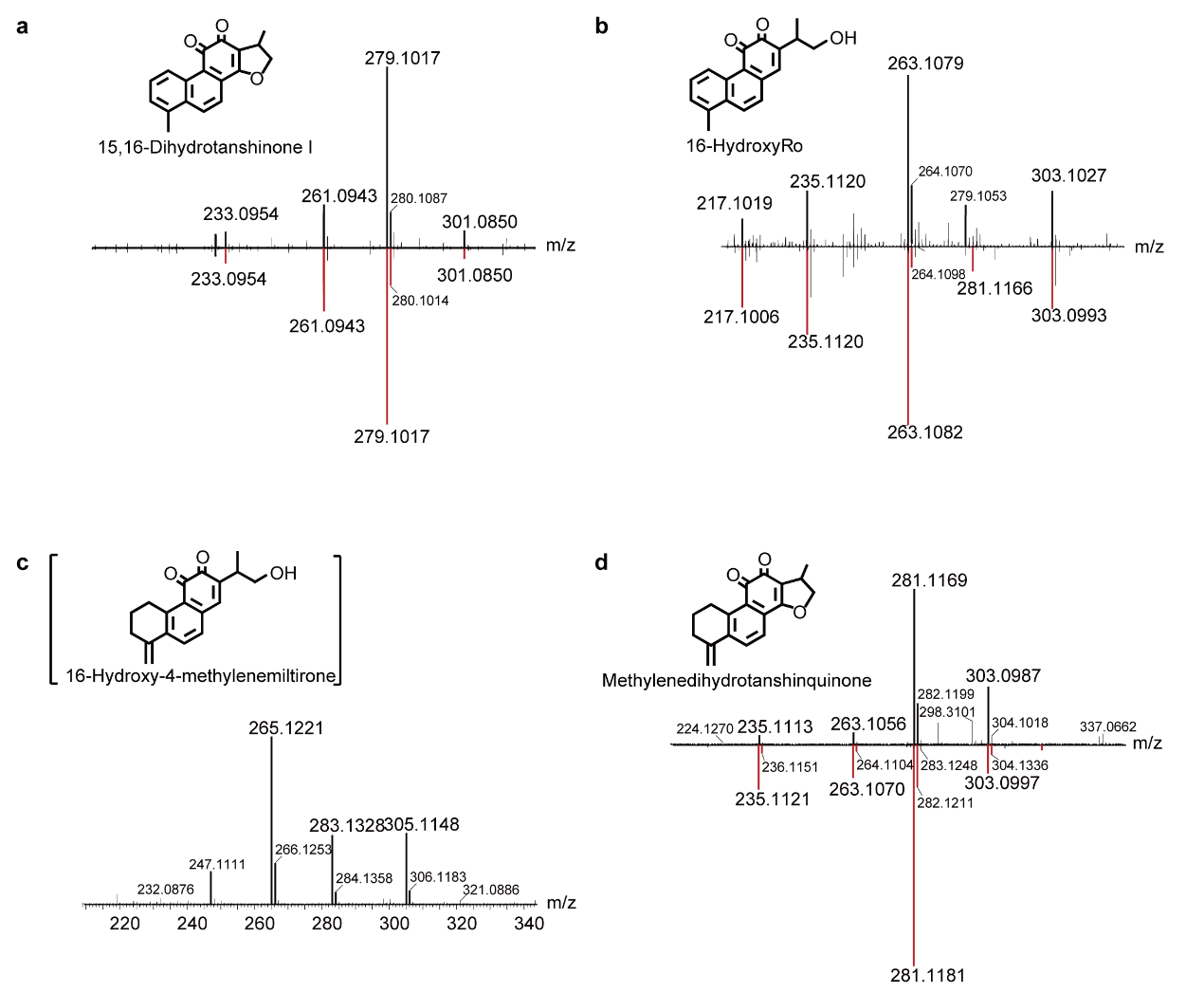
**

**Supplementary Fig. 7 Identification of the CYP71D375 products from compounds 5 and 6.**

Butterfly plots of mass spectra for **(a)** product **2** and authentic standard for 15,16-dihydrotanshinone I, both with retention time (RT) = 2.50 min; (**b**) product **9** and authentic standard for 16-hydroxyRo, both with RT = 1.48 min; along with mass spectrum for (**c**) product **10**, with RT = 1.78 min and (**d**) product **11**, with RT = 2.85 min. Enzyme reaction products are colored black and the standards are colored red in each case.


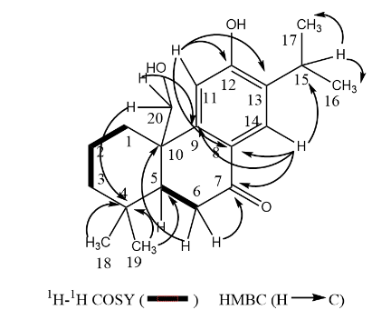
**a**

**b**

**c**

**d**

**e**

**f**

**Supplementary Fig. 8 Structural characterization of 20-hydroxysugiol (12) by NMR.**

Chemical shift assignments, based on (**a**) assigned HMBC and COSY correlations, (**b**) ^1^H NMR spectrum in CH_3_DO, (**c**) ^13^C NMR spectrum in CH_3_DO, (**d**) HSQC spectrum in CH_3_DO, (**e**) HH in CH_3_DO COSY spectrum, (**f**) HMBC spectrum in CH_3_DO.

^1^H NMR data (600 MHz, CH_3_DO): δ 7.83 (1H, s, H-14), 6.79 (1H, s, H-11), 3.91 (1H, d, *J* = 11.3 Hz, Ha-20), 3.81 (1H, d, *J* = 11.3 Hz, Hb-20), 3.23 (1H, m, H-15), 2.78 (1H, dd, *J* = 18.4; 14.4 Hz, Ha-6), 2.54 (1H, dd, *J* = 18.4; 4.5 Hz, Hb-6), 1.95 (1H, dd, *J* = 14.4; 4.5 Hz, H-5), 1.80 (1H, m, Ha-2), 1.66 (1H, m, Hb-2), 1.58 (1H, dd, *J* = 13.8; 1.2 Hz, Ha-3), 1.35 (1H, m, Hb-3), 1.32 (2H, m, H-1), 1.21 (3H, d, *J* = 2.0 Hz, H-16), 1.20 (3H, d, *J* = 2.0 Hz, H-17), 1.05 (3H, s, H-18), 0.96 (3H, s, H-19)；^13^C NMR data (150 MHz, CH_3_DO): δ 201.2 (C-7), 161.5 (C-12), 153.6 (C-9), 134.8 (C-13), 127.0 (C-14), 125.5 (C-8), 113.5 (C-11), 65.2 (C-20), 51.4 (C-5), 44.1 (C-10), 42.6 (C-3), 36.6 (C-6), 34.2 (C-4), 33.2 (C-19), 30.9 (C-1), 27.9 (C-15), 22.8 (C-17), 22.8 (C-16), 22.4 (C-18), 19.8 (C-2).

**
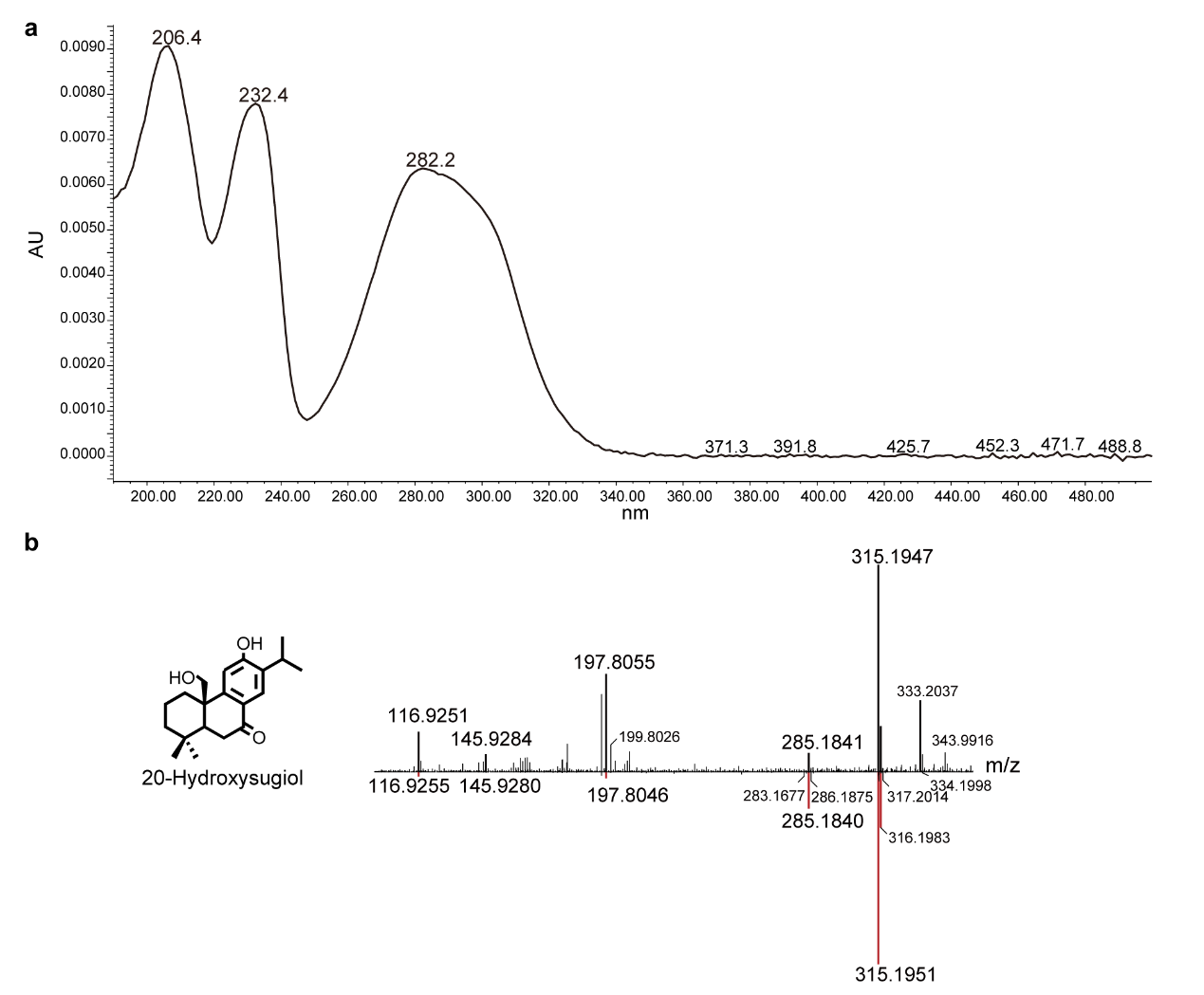
**

**Supplementary Fig. 9 Butterfly plots of mass spectra for product 12 (black) and NMR characterized 20-hydroxysugiol standard (red), m/z 315.1951 [M-H]^-^, both with RT = 2.45 min.**

**

**

**Supplementary Fig. 10 Proposed reaction mechanism for the cyclization of miltirone to cryptotanshinone.**


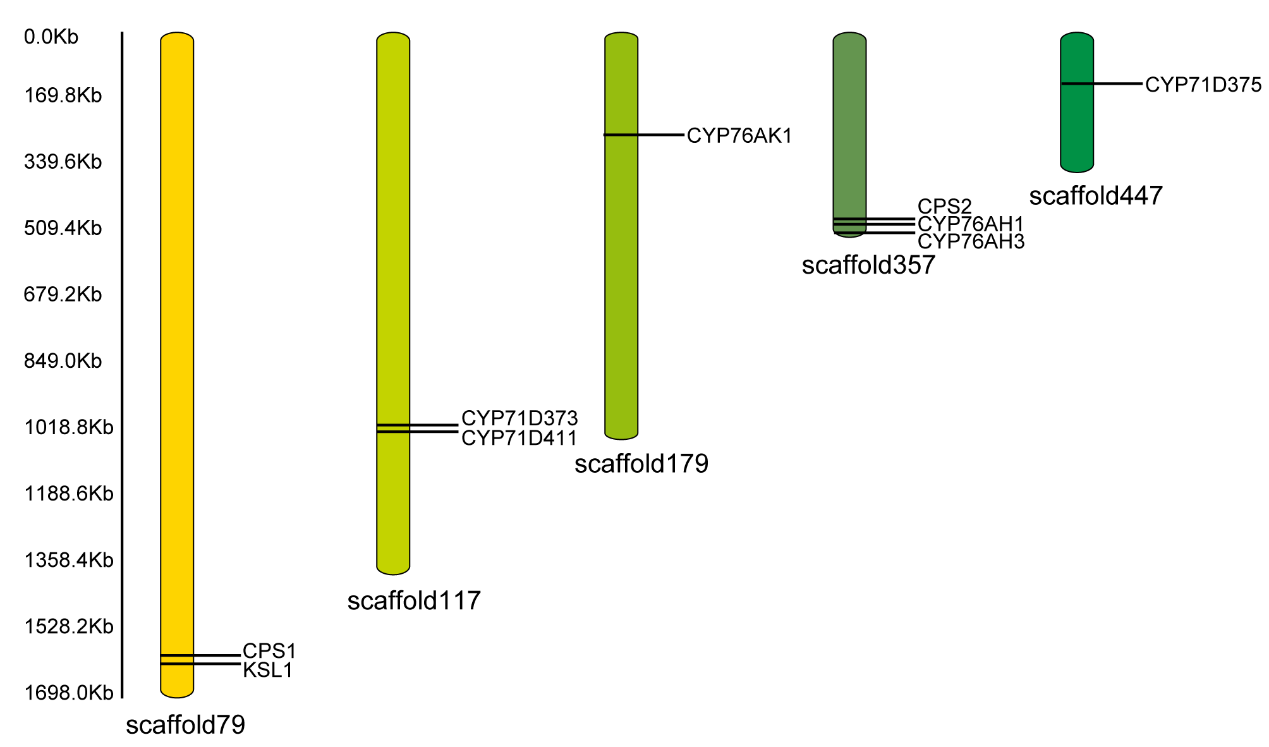


**Supplementary Fig. 11 The positions of nine identified genes on scaffolds.**


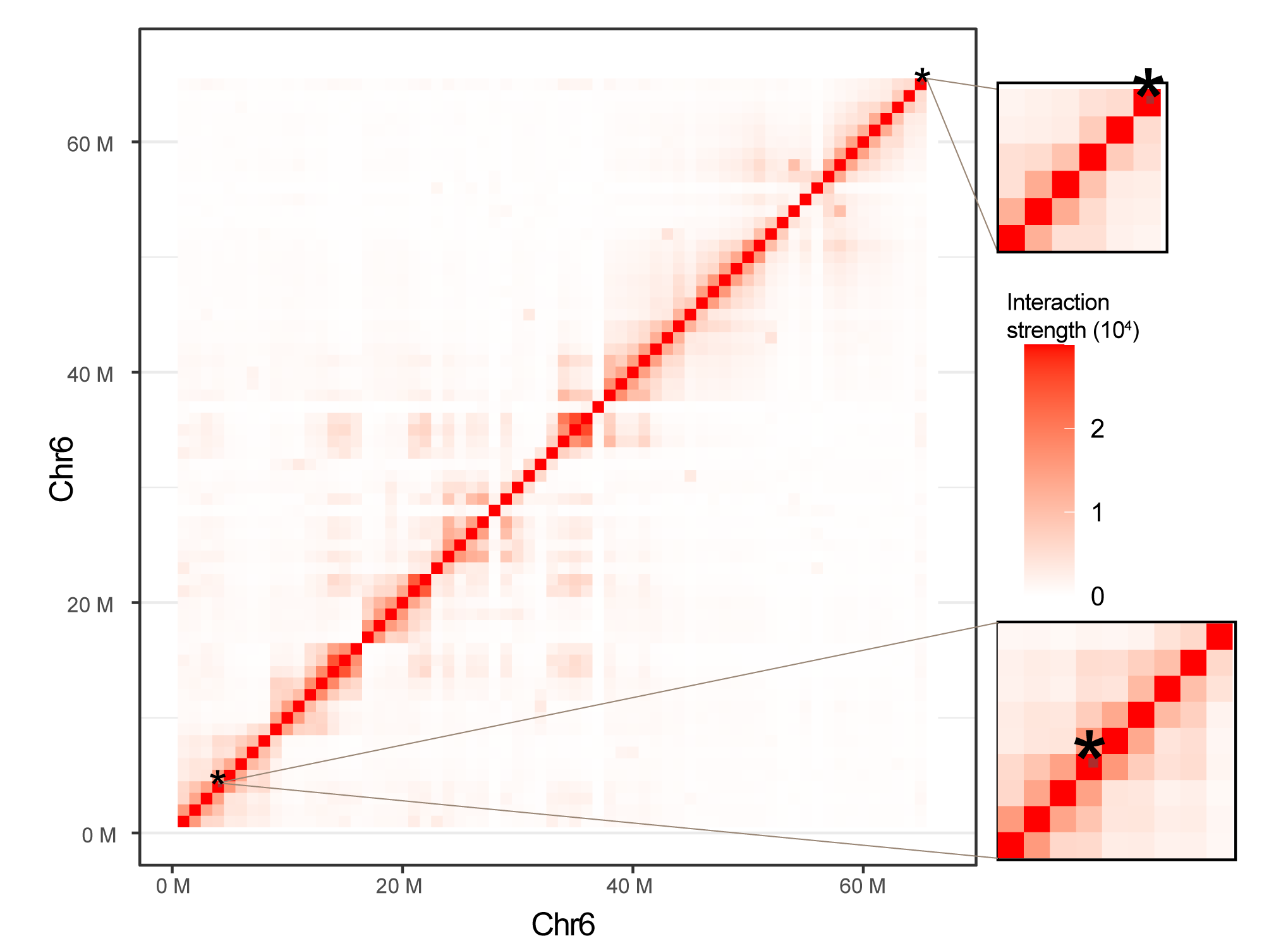


**Supplementary Fig. 12** **Interaction heat-map of pseudochromosome 6 based on Hi-C analysis.** The asterisks show ferruginol biosynthetic gene cluster (lower left) and CYP71D tandem gene array (upper right). Source data are provided in Source Data file.

*


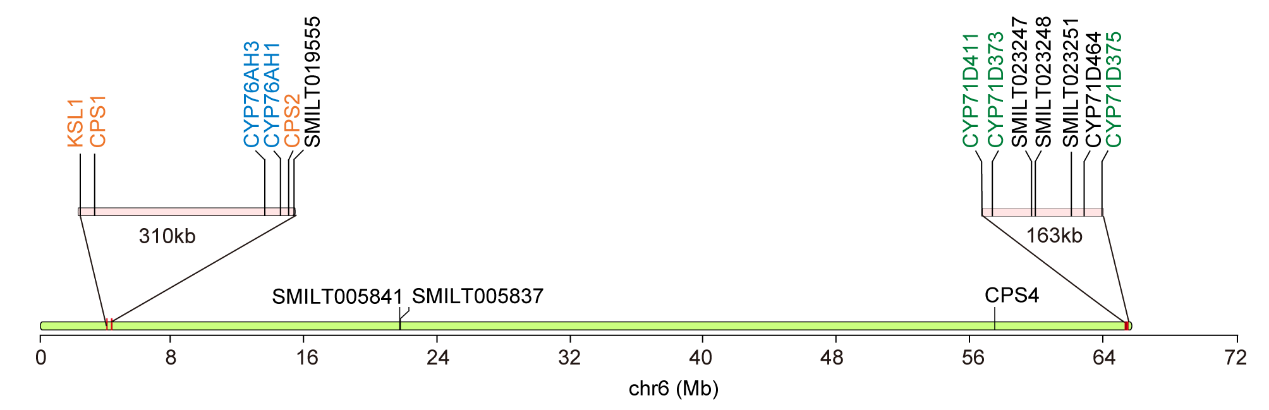


**Supplementary Fig. 13 Gene location of identified diTPS and CYP450 genes in pseudochromosome 6. Genes in orange, blue and green are functional genes.**

**
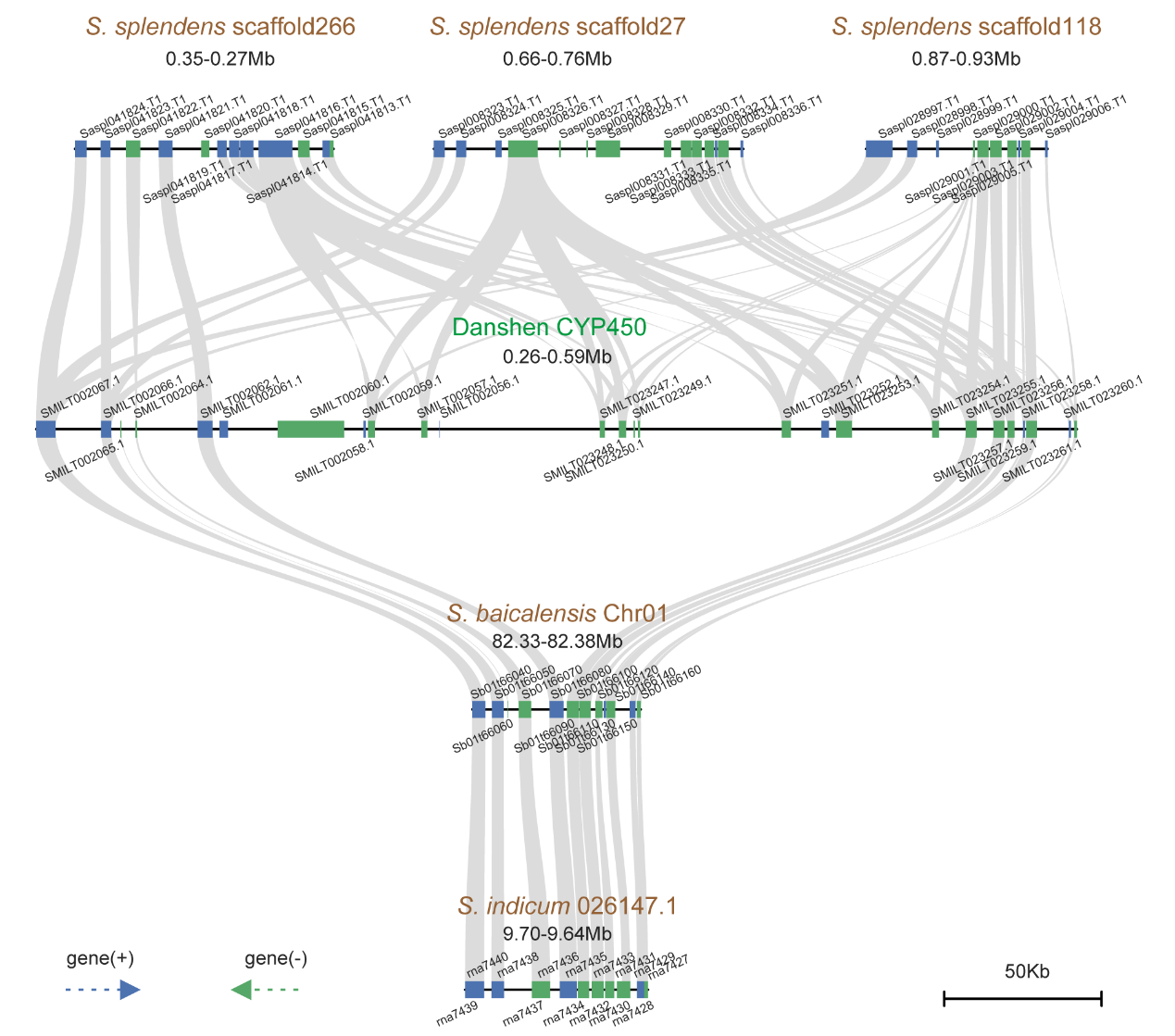
**

**Supplementary Fig. 14 Syntenic analysis of CYP71D genes in Danshen, *S. splendens*, *S. baicalensis* and *S. indicum.*** Source data are provided in Source Data file.

**Supplementary Tables**

**Supplementary Table 1 Clean data statistics of different insert size libraries used in genome assembly**

| **Pair-end Libraries** | **Insert Size** | **Average Reads Length (bp)** | **Total Data (Gb)** | **Sequence**  **Depth (X)** |
| --- | --- | --- | --- | --- |
| Solexa Reads | 200 bp | 125 | 18.83 | 29.88 |
|  | 450 bp | 250 | 67.34 | 106.88 |
|  | 500 bp | 125 | 81.59 | 129.5 |
|  | 800 bp | 125 | 13.26 | 21.04 |
|  | 2 kb | 100 | 26.79 | 42.53 |
|  | 5 kb | 110 | 81.05 | 128.65 |
|  | 10 kb | 110 | 40.75 | 64.68 |
|  | 20 kb | 100 | 12.08 | 19.18 |
| Total | - | - | 341.69 | 542.36 |

**Supplementary Table 2 Clean data statistics of PacBio libraries used in genome assembly**

| **Insert Size** | **Average Length (kb)** | **Total Data (Gb)** | **N50 (kb)** | **Max Length (kb)** | **Sequence Depth (X)** |
| --- | --- | --- | --- | --- | --- |
| 20 k | 4.40 | 7.43 | 10,463 | 47,183 | 12.38 |
| 20 k | 7.63 | 22.7 | 11,792 | 51,531 | 37.83 |
| Total | 6.46 | 30.13 | 11,519 | 51,531 | 50.21 |

**Supplementary Table 3 Summary of Danshen genome assembly**

|  | **Contig** | | **Scaffold** | |
| --- | --- | --- | --- | --- |
|  | **Size (bp)** | **Number** | **Size (bp)** | **Number** |
| **N90** | 127,078 | 1,151 | 335,574 | 462 |
| **N80** | 216,340 | 820 | 548,808 | 337 |
| **N70** | 296,774 | 606 | 762,573 | 250 |
| **N60** | 385,276 | 441 | 1,019,969 | 188 |
| **N50** | 505,211 | 314 | 1,261,664 | 139 |
| **Longest** | 5,474,769 |  | 5,474,769 |  |
| **Total Size** | 554,060,280 |  | 557,506,879 |  |
| **Total Number (>100 bp)** |  | 3,923 |  | 1,115 |
| **Total Number (>2 kb)** |  | 3,424 |  | 1,022 |

**Supplementary Table4 Evaluation of coverage by reads mapping**

| **Total Bases** | **Total Reads** | **All Coverage (%)** | **All Depth (X)** | **Uniq Coverage (%)** | **Uniq Depth (X)** |
| --- | --- | --- | --- | --- | --- |
| 557,506,879 | 1,178,962,980 | 97.87 | 269.38 | 91.53 | 228.41 |

**Supplementary Table 5 BUSCO evaluation of the assembly**

| Software | Total genes | **Complete** | | | **Fragmented** | **Missing** |
| --- | --- | --- | --- | --- | --- | --- |
|  |  | Total | Single-copy | **Duplicated** |  |  |
| *BUSCO* | 1,440 | 1,312 (91.10%） | 1,215 (84.40%) | 97 (6.70%) | 30 (2.10%) | 98 (6.80%) |

**Supplementary Table 6 Evaluation of coverage by mapping ESTs**

| **Dataset** | **Number** | **Total  length (bp)** | **Bases covered by**  **assembly (%)** | **Sequences covered by**  **assembly (%)** | **with >90% sequence in one Scaffold** | | **with >50% sequence in one Scaffold** | |
| --- | --- | --- | --- | --- | --- | --- | --- | --- |
|  |  |  |  |  | **Number** | **Percent** | **Number** | **Percent** |
| >0 bp | 133,717 | 190,147,171 | 93.83 | 95.98 | 115,332 | 86.25 | 126,191 | 94.37 |
| >200 bp | 133,717 | 190,147,171 | 93.83 | 95.98 | 115,332 | 86.25 | 126,191 | 94.37 |
| >500 bp | 92,758 | 176,395,155 | 94.24 | 98.20 | 79,734 | 85.96 | 89,529 | 96.52 |
| >1000 bp | 64,534 | 156,282,674 | 94.37 | 99.56 | 54,782 | 84.89 | 62,938 | 97.53 |

**Supplementary Table 7 Gene prediction in the Danshen genome**

| **Gene set** |  | **Number** | **Average transcript length (bp)** | **Average CDS length (bp)** | **Average exon per gene** | **Average exon length (bp)** | **Average intron length (bp)** |
| --- | --- | --- | --- | --- | --- | --- | --- |
| ***De novo*** | ***AUGUSTUS*** | 40,894 | 2086.79 | 962.86 | 4.25 | 226.77 | 346.26 |
|  | ***GENSCAN*** | 35,409 | 8908.67 | 1111.12 | 5.34 | 207.97 | 1795.52 |
|  | ***GlimmerHMM*** | 42,787 | 1653.18 | 794.76 | 3.32 | 239.84 | 369.98 |
| ***Homolog*** | ***A.thaliana*** | 42,102 | 1762.81 | 858.64 | 2.95 | 290.57 | 462.5 |
|  | ***D.hygrometricum*** | 41,853 | 2456.83 | 1138.68 | 3.17 | 358.82 | 606.48 |
|  | ***S.indicum*** | 34,819 | 3174.48 | 1310.53 | 3.99 | 328.77 | 624.2 |
|  | ***S.lycopersicum*** | 49,457 | 2084.26 | 972.06 | 2.99 | 324.7 | 557.85 |
|  | ***S.tuberosum*** | 31,252 | 4001.51 | 1256.24 | 3.86 | 325.87 | 961.54 |
|  | ***U.gibba*** | 33,693 | 2674.18 | 1161.7 | 3.43 | 338.92 | 623.03 |
| ***Consensus*** | ***GLEAN*** | 36,037 | 2599.02 | 1006.55 | 4.19 | 240.13 | 498.95 |
| **Transcriptome** | **RNA-Seq leaf** | 19,421 | 3261.6 | 1001.14 | 4.13 | 242.39 | 403.43 |
|  | **RNA-Seq root** | 19,320 | 3750.95 | 1142.41 | 5.02 | 227.42 | 390.54 |
|  | **RNA-Seq flower** | 14,010 | 2722.47 | 867.86 | 3.81 | 227.51 | 388.67 |
| **Final set** * |  | 33,760 | 2771.39 | 1050.92 | 4.36 | 240.92 | 459.45 |

* Genes with more than 10% ambiguous bases in CDS region have been filtered.

**Supplementary Table 8 Summary of Danshen gene annotation based on homology or functional classification**

|  | | | **Number** | **Percent (%)** |
| --- | --- | --- | --- | --- |
| **Total** | |  | 33,760 | 100 |
| **Annotated** | **InterPro**  **GO**  **KEGG**  **Nr**  **COG**  **Swissprot**  **TrEMBL** | | 22,898 | 67.83 |
|  |  |  | 17,058 | 50.53 |
|  |  |  | 15,214 | 45.07 |
|  |  |  | 27,910 | 82.67 |
|  |  |  | 10,440 | 30.92 |
|  |  |  | 19,902 | 58.95 |
|  |  |  | 27,672 | 81.97 |
| **Annotated** | |  | 28,063 | 83.13 |
| **Unannotated** | |  | 5,697 | 16.87 |

**Supplementary Table 9 Noncoding genes in the Danshen genome**

| **Type** | | **Copy** | **Average length (bp)** | **Total length (bp)** | **% of genome** |
| --- | --- | --- | --- | --- | --- |
| **miRNA** |  | 129 | 119.64 | 15,434 | 0.0028 |
| **tRNA** |  | 682 | 75.14 | 51,248 | 0.0094 |
| **rRNA** | rRNA | 282 | 240.46 | 68,093 | 0.0124 |
|  | 18S | 58 | 681.22 | 39,511 | 0.0072 |
|  | 28S | 81 | 139.16 | 11,272 | 0.0021 |
|  | 5.8S | 20 | 154.15 | 3,083 | 0.0006 |
|  | 5S | 123 | 115.67 | 14,227 | 0.0025 |
| **snRNA** | snRNA | 844 | 112.4 | 94,864 | 0.0173 |
|  | CD-box | 625 | 103.51 | 64,694 | 0.0118 |
|  | HACA-box | 84 | 130.88 | 10,994 | 0.002 |
|  | splicing | 135 | 142.04 | 19,176 | 0.0035 |

**Supplementary Table 10 Repeat elements in Danshen genomes**

|  | RepBase TEs | | TE Proteins | | *De novo* | | Combined TEs | |
| --- | --- | --- | --- | --- | --- | --- | --- | --- |
|  | Length  (bp) | % in Genome | Length  (bp) | % in Genome | Length  (bp) | % in Genome | Length  (bp) | % in  Genome |
| **DNA** | 7,704,330 | 1.41 | 9,865,608 | 1.80 | 31,082,342 | 5.68 | 40,777,511 | 7.45 |
| **LINE** | 3,609,982 | 0.66 | 14,859,157 | 2.71 | 11,817,355 | 2.16 | 23,998,875 | 4.38 |
| **SINE** | 6,940 | 0.001 | 0 | 0.00 | 134,695 | 0.02 | 141,306 | 0.03 |
| **LTR** | 41,274,845 | 7.54 | 66,886,973 | 12.21 | 249,812,531 | 45.63 | 253,473,996 | 46.30 |
| **Other** | 32,338 | 0.06 | 0.00 | 0.00 | 0.00 | 0.00 | 32,338 | 0.006 |
| **Unknown** | 40,385 | 0.007 | 2,649 | 0.0005 | 6,513,161 | 1.19 | 6,555,461 | 1.20 |
| **Total** | 52,229,070 | 9.54 | 91,606,858 | 16.73 | 291,980,130 | 53.33 | 308,033,395 | 56.27 |

**Supplementary Table 11 Homology matrix of the eight CYP71Ds in clade I in Fig. 3a**

| SMILT002058 | 100% |  |  |  |  |  |  |  |
| --- | --- | --- | --- | --- | --- | --- | --- | --- |
| SMILT002057 | 83.0% | 100% |  |  |  |  |  |  |
| SMILT023253 | 84.9% | 85.8% | 100% |  |  |  |  |  |
| SMILT023254 | 82.1% | 87.4% | 86.5% | 100% |  |  |  |  |
| SMILT023247 | 82.3% | 90.4% | 85.1% | 86.2% | 100% |  |  |  |
| SMILT023248 | 83.7% | 89.4% | 86.7% | 86.2% | 90.6% | 100% |  |  |
| SMILT023251 | 81.0% | 84.9% | 88.5% | 86.1% | 82.6% | 83.7% | 100% |  |
| SMILT019555 | 70.9% | 73.9% | 69.5% | 71.7% | 71.6% | 72.5% | 73.3% | 100% |

**Supplementary Table 12 Genome-wide blast of the RNAi target**

| **Sequences producing significant alignments** | **Score（bits）** | **E Value** |
| --- | --- | --- |
| SMILT002058.1 [mRNA] locus=scaffold117:1019103:1021337:+ | 898 | 0.0 |
| SMILT023253.1 [mRNA] locus=scaffold447:97385:102373:- | 363 | e-100 |
| SMILT023248.1 [mRNA] locus=scaffold447:28280:30611:- | 325 | 1e-088 |
| SMILT002057.1 [mRNA] locus=scaffold117:1002451:1004351:+ | 293 | 5e-79 |
| SMILT023254.1 [mRNA] locus=scaffold447:127820:130045:- | 266 | 1e-070 |
| SMILT023247.1 [mRNA] locus=scaffold447:22198:23854:- | 260 | 7e-69 |
| SMILT023251.1 [mRNA] locus=scaffold447:80099:82961:- | 208 | 2e-053 |
| SMILT023249.1 [mRNA] locus=scaffold447:32970:33362:- | 121 | 4e-027 |
| SMILT011389.1 [mRNA] locus=scaffold214:150629:153478:+ | 36 | 0.20 |
| SMILT031033.1 [mRNA] locus=scaffold78:508706:511614:- | 36 | 0.20 |
| SMILT025978.1 [mRNA] locus=scaffold53:122652:124932:- | 36 | 0.20 |
| SMILT017935.1 [mRNA] locus=scaffold32:1051662:1054844:+ | 36 | 0.20 |
| SMILT019555.1 [mRNA] locus=scaffold357:467200:469728:+ | 34 | 0.77 |
| SMILT017004.1 [mRNA] locus=scaffold300:354622:356990:+ | 34 | 0.77 |
| SMILT004494.1 [mRNA] locus=scaffold138:1124808:1136468:- | 34 | 0.77 |
| SMILT033305.1 [mRNA] locus=scaffold94:518094:523585:+ | 34 | 0.77 |
| SMILT023732.1 [mRNA] locus=scaffold46:1491985:1499242:+ | 34 | 0.77 |
| SMILT027868.1 [mRNA] locus=scaffold6:3767283:3771281:- | 34 | 0.77 |

**Supplementary Table 13 The gene expression level of down and up regulated genes involved in *CYP71Ds*-RNAi lines compared with WT.** Source data are provided in Source Data file.

| **Type** | **Gene** | **FPKM** | | | **Annotation** |
| --- | --- | --- | --- | --- | --- |
|  |  | **WT** | | **RNAi** |  |
| downregulated genes | CYP71D411 | 565.06 | 14.49 | |  |
|  | CYP71D373 | 86.07 | 5.70 | |  |
|  | CYP71D375 | 147.48 | 12.08 | |  |
|  | CYP71D464 | 124.16 | 12.16 | |  |
|  | CPS1 | 312.10 | 192.85 | |  |
|  | KSL1 | 111.63 | 50.62 | |  |
|  | CYP76AH1 | 778.48 | 437.36 | |  |
|  | CYP76AH3 | 552.67 | 236.27 | |  |
|  | CYP76AK1 | 181.78 | 98.59 | |  |
|  | SMILT019558.1 | 520.58 | 220.88 | | SmCYP76AH29P |
|  | SMILT031681.1 | 52.29 | 14.36 | | SmCYP71AU 66 |
|  | SMILT027710.1 | 97.64 | 45.34 | | terpene synthase 7-like |
|  | SMILT026335.1 | 542.83 | 210.78 | | PREDICTED: 1-aminocyclopropane-1-carboxylate oxidase homolog 1-like |
|  | SMILT024848.1 | 164.47 | 76.02 | | geranyl diphosphate synthase small subunit type II.1 |
|  | SMILT033381.1 | 73.69 | 34.42 | | uncharacterized protein |
|  | SMILT009153.1 | 85.63 | 40.56 | | SmCYP71BE51 |
|  | SMILT025363.1 | 38.78 | 18.76 | | PREDICTED: potassium transporter 5 |
| upregulated genes | SMILT023100.1 | 8.63 | 22.69 | | uncharacterized protein |
|  | SMILT011017.1 | 1.23 | 4.41 | | SmCYP81B76 |
|  | SMILT003521.1 | 3.12 | 6.86 | | uncharacterized protein |
|  | SMILT001293.1 | 0.81 | 5.21 | | SmCYP71D414 |
|  | SMILT031890.1 | 2.30 | 9.36 | | uncharacterized protein |
|  | SMILT032012.1 | 32.16 | 106.20 | | unknown |
|  | SMILT003352.1 | 15.63 | 51.56 | | allergen |
|  | SMILT003265.1 | 11.96 | 58.54 | | putative germin-like protein |
|  | SMILT020329.1 | 13.74 | 60.41 | | uncharacterized protein |
|  | SMILT030763.1 | 106.12 | 279.43 | | SMLII |
|  | SMILT006355.1 | 7.95 | 28.57 | | PREDICTED: E3 ubiquitin ligase BIG BROTHER |
|  | SMILT029837.1 | 6.46 | 14.82 | | PREDICTED: probable LRR receptor-like serine/threonine-protein kinase |
|  | SMILT005525.1 | 31.24 | 65.05 | | PREDICTED: glutamine synthetase cytosolic isozyme isoform X1 |
|  | SMILT001757.1 | 17.21 | 39.64 | | SmCYP76AK5 |
|  | | | | | |

**Supplementary Table 14 Primers used in this study**

| **Name** | **Sequence (5' to 3')** |
| --- | --- |
| **Full length cloning primers** |  |
| *CYP71D3*73-FF | TTGCGGCCGCAAATGGAGTTTAACATCTCATCTACACTCA |
| *CYP71D373*-FR | GACTAGTCTCAAGCAGCTGCGGACAAAGGCCGCTTA |
| *CYP71D375*-FF | TTGCGGCCGCAAATGGAGTTCAACATCCCATCAACACTCA |
| *CYP71D375*-FR | GACTAGTCTCAAGCAGCTGCACGCAAAGGTCGTTTA |
| *CYP71D411*-FF | TTGCGGCCGCAAATGAAATCTCACATCATGGAGCTA |
| *CYP71D411*-FR | GACTAGTCTCACGCACGCAAAGGCCGCTTC |
| *CYP71D464*-FF | TTGCGGCCGCAAATGGTAGGCGCCGCCTTGCCCCAC |
| *CYP71D464*-FR | GACTAGTCCTATCTTGCAGGAAGAGGTCGCTTCAC |
| **Primers for GATEWAY RNA interference construction** |  |
| *CYP71D411*-823F | TGGATGTTCTCCTTCAGATTCA |
| *CYP71D411*-1276R | CTTTGAAATCAACGGCGCTTTCCTCA |
| **Primers for CYP71D375 mutation** |  |
| His-*CYP71D375*-F | CAAGGAGAAAAAACCCCGATGGAGTTCAACATCCCATCAAC |
| His-*CYP71D375*-R | AGTGAGTCGTATTACGGATCTCAAGCAGCTGCACGCAAAG |
| I300L-F | GTGCTGGATATGTTCCTTGCAGGAAC |
| I300L-R | GTTCCTGCAAGGAACATATCCAGCAC |
| I300S-F | GTGCTGGATATGTTCTCTGCAGGAAC |
| I300S-R | AGAGAACATATCCAGCACCACCGCTTTG |
| I300F-F | GTGCTGGATATGTTCTTTGCAGGAAC |
| I300F-R | AAAGAACATATCCAGCACCACCGCTTTG |
| I300T-F | GTGCTGGATATGTTCACTGCAGGAAC |
| I300T-R | GTTCCTGCAGTGAACATATCCAGCAC |
| I300M-F | GGTGCTGGATATGTTCATGGCAGGAAC |
| I300M-R | GTTCCTGCCATGAACATATCCAGCACC |
| I300A-F | GTGCTGGATATGTTCGCTGCAGGAAC |
| I300A-R | AGCGAACATATCCAGCACCACCGCTTTG |
| I300G-F | GGTGCTGGATATGTTCGGTGCAGGAAC |
| I300G-R | TCAGTTCCTCCVAATGAACATATCCAG |
| I300V-F | GGTGCTGGATATGTTCGTGGCAGGAAC |
| I300V-R | GTTCCTGCCACGAACATATCCAGCACC |
| A301G-F | CTGGATATGTTCATTGGAGGAACTGA |
| A301G-R | TCAGTTCCTCCAATGAACATATCCAG |
| A301S-F | CTGGATATGTTCATTTCAGGAACTGA |
| A301S-R | TCAGTTCCTGAAATGAACATATCCAG |
| A301F-F | CTGGATATGTTCATTTTCGGAACTGA |
| A301F-R | TCAGTTCCGAAAATGAACATATCCAG |
| A301T-F | CTGGATATGTTCATTACAGGAACTGA |
| A301T-R | TCAGTTCCTGTAATGAACATATCCAG |
| A300I-F | CTGGATATGTTCATTATAGGAACTGA |
| A300I-R | TCAGTTCCTATAATGAACATATCCAG |
| A301H-F | CTGGATATGTTCATTCACGGAACTGA |
| A301H-R | TCAGTTCCGTGAATGAACATATCCAG |
| L366F-F | TGAGATTGCACCCACCGTTCCCGTTTCT |
| L366F-R | AGAAACGGGAACGGTGGGTGCAATCTCA |
| L366T-F | TGAGATTGCACCCACCGACGCCGTTTCT |
| L366T-R | AGAAACGGCGTCGGTGGGTGCAATCTCA |
| L366I-F | TGAGATTGCACCCACCGATACCGTTTCT |
| L366I-R | AGAAACGGTATCGGTGGGTGCAATCTCA |
| L366N-F | TGAGATTGCACCCACCGAATCCGTTTCT |
| L366N-R | AGAAACGGATTCGGTGGGTGCAATCTCA |
| L366A-F | TGAGATTGCACCCACCGGCGCCGTTTCT |
| L366A-R | AGAAACGGCGCCGGTGGGTGCAATCTCA |
| L366R-F | GATTGCACCCACCGAGGCCGTTTCTAGTC |
| L366R-R | GACTAGAAACGGCCTCGGTGGGTGCAATC |
| L366G-F | GCACCCACCGGGGCCGTTTCTAGTCCCCAG |
| L366G-R | CTGGGGACTAGAAACGGCCCCGGTGGGTGC |
| L366V-F | GCACCCACCGGTGCCGTTTCTAGTCCCCAG |
| L366V-R | CTGGGGACTAGAAACGGCCACGGTGGGTGC |
| V370F-F | CCCACCGTTGCCGTTTCTATTCCCCAGAAT |
| V370F-R | ATTCTGGGGAATAGAAACGGCAACGGTGGG |
| V370T-F | CCCACCGTTGCCGTTTCTAACCCCCAGAAT |
| V370T-R | ATTCTGGGGGTTAGAAACGGCAACGGTGGG |
| V370I-F | CCCACCGTTGCCGTTTCTAATCCCCAGAAT |
| V370I-R | ATTCTGGGGATTAGAAACGGCAACGGTGGG |
| V370L-F | CCCACCGTTGCCGTTTCTACTCCCCAGAAT |
| V370L-R | ATTCTGGGGAGTAGAAACGGCAACGGTGGG |
| V370A-F | CCCACCGTTGCCGTTTCTAGCCCCCAGAAT |
| V370A-R | ATTCTGGGGGCTAGAAACGGCAACGGTGGG |
| V370R-F | TTGCCGTTTCTACGCCCCAGAATCAAC |
| V370R-R | GTTGATTCTGGGGCGTAGAAACGGC |
| V370W-F | GTTGCCGTTTCTATGGCCCAGAATCAACG |
| V370W-R | CGTTGATTCTGGGCCATAGAAACGGCAAC |
| V370Q-F | CGTTGCCGTTTCTACAGCCCAGAATCAACG |
| V370Q-R | CGTTGATTCTGGGCTGTAGAAACGGCAACG |
| V482F-F | ATATGGCGGAGGCCTTCGGTTTCACCGTTAG |
| V482F-R | CTAACGGTGAAACCGAAGGCCTCCGCCATAT |
| V482T-F | ATATGGCGGAGGCCTTCGGTACCACCGTTAG |
| V482T-R | CTAACGGTGGTACCGAAGGCCTCCGCCATAT |
| V482I-F | ATATGGCGGAGGCCTTCGGTATCACCGTTAG |
| V482I-R | CTAACGGTGATACCGAAGGCCTCCGCCATAT |
| V482S-F | ATATGGCGGAGGCCTTCGGTTCCACCGTTAG |
| V482S-R | CTAACGGTGGAACCGAAGGCCTCCGCCATAT |
| V482A-F | ATATGGCGGAGGCCTTCGGTGCCACCGTTAG |
| V482A-R | CTAACGGTGGCACCGAAGGCCTCCGCCATAT |
| V482M-F | GGCCTTCGGTATGACCGTTAGAAAGAAAC |
| V482M-R | GTTTCTTTCT AACGGTCATA CCGAAGGCC |
| V482W-F | GGCCTTCGGTTGGACCGTTAGAAAG |
| V482W-R | CTTTCTAACGGTCCAACCGAAGGCC |
| V482K-F | GGCCTTCGGTAAGACCGTTAGAAAGAAAC |
| V482K-R | GTTTCTTTCTAACGGTCTTACCGAAGGCC |
| V482I-F | GCCTTCGGTATCACCGTTAGAAAGAAAC |
| V482I-R | GTTTCTTTCTAACGGTGATACCGAAGGC |
| V482L-F | GCCTTCGGTCTCACCGTTAGAAAGAAAC |
| V482L-R | GTTTCTTTCTAACGGTGAGACCGAAGGC |

**Supplemental Methods**

**Supplemental Method 1. Genome assembly**

Two intermediate assemble versions for the genome were generated using Illumina reads (v0.1) by DISCOVAR and PacBio reads (v0.2) by Falcon (v1.7.4), separately. Then they were merged together using the HABOT (hybrid assembly of third-generation sequencing 2; https://github.com/asarum/HABOT2) software (1gene Corp., Hangzhou, China)[^1^](#_ENREF_1). To combine contigs from different versions of assembly and construct a new contig sets, HABOT with the following four modules were used. The first one is Graph module. This module counts k-mer frequencies and extracts the unique k-mers from Illumina reads. A unique k-mer is theoretically defined as k-bp sequences that occur just once in a haploid genome and is calculated following a Poisson model as previously described. Using unique k-mers, instead of all the k-mers, for graph construction minimizes the effects of error-prone repeats and increases computation speed. The second one is align module. This module is use for an all-to-all alignment between PacBio contigs and Illumina contigs. By using unique k-mers it performs the alignment much faster than BLASR and is of high accuracy. The third one is duplication remove module. When two sequences have common unique k-mers that exceed a cutoff (default is 0.5), the shorter sequence is removed. The last module is de novo module. This module calls the above 3 modules and performs hybrid assembly.

For the assembly, we extracted unique 17-mers from the two PCR-free Illumina libraries reads. Overlaps among contigs from different intermediate assembly versions were identified using the alignment module. Then the OLC graph was built by the overlap contigs. The connection is dropped in case of the following situation: (1) contig A’s best connection is contig B; (2) contig B’s best connection is contig C; (3) give up connection from A to B if A has no alignment with C. Afterwards duplicated regions in the contig set were removed. Finally scaffolding and gap closure were performed on the new contig set using Illumina mate pair reads with SSPACE v3.0 and GapCloser v1.12 (both with default parameters) to generate assembly.

**Supplemental Method 2. Genome Annotation**

Repeat sequences of *Salvia miltiorrhiza* genome were identified with a combination of *de novo* and homolog strategies. RepeatMasker (v4.0.3) and RepeatProteinMask (3.3.0) (<http://www.repeatmasker.org>) software were employed to find the transposons components based on the RepBase library (<http://www.girinst.org/repbase>) with an e-value cutoff 1e^-5^. TRF (4.07b)[^2^](#_ENREF_2) software was used to identify tandem repeats. RepeatModeler (v1.0)[^3^](#_ENREF_3) and LTR-FINDER (v1.05)[^4^](#_ENREF_4) were used to identify *de novo* repeat sequences in the assemble genome. Finally, the identified repeat sequences were used to construct a non-redundant repeat sequence library, and repeat sequences with an identity more than 50% were grouped into the same classes.

The gene prediction pipeline of the *Salvia miltiorrhiza* genome combined *ab initio* gene prediction, homologous sequence searching and transcriptome sequence mapping. GlimmerHMM (v1.1.0) (http://ccb.jhu.edu/software/glimmerhmm/) and Augustus (v3.0.2) (http://bioinf.uni-greifswald.de/augustus/) were used to carry out *de novo* prediction. For homology-based gene prediction, we aligned *Arabidopsis thaliana*, *Capsicum annuum*, *Solanum lycopersicum*, *Solanum tuberosum*, *Boea hygrometrica*, *Mimulus gutatus*, *Andrographis paniculata*, *Sesamum indicum*, *Utricularia gibba,* *S. splendens,* and *Scutellaria barcalensis* protein sequences to the genome by using BLASTX (http://blast.ncbi.nlm.nih.gov/Blast.cgi) with parameters of e-value cutoff of 1e^-5^. Then GeneWise^[5](#_ENREF_5)^ was employed for the further alignments to extract accurate exon-intron information. GLEAN(v1.1)[^6^](#_ENREF_6) was used to merge *de novo* gene sets, homology-based gene sets into the consensus gene set. The RNA-seq reads generated from Root, Leaves and Flowers were used for gene annotation. The TopHat^[7](#_ENREF_7)^ were used to align the RNA-Seq reads to the assembly genome to identify the accurate spice junctions between each exons, then the Cufflinks (v2.2.1)[^8^](#_ENREF_8) were used to get a set of assembled transcripts by integrate with other evidence.

Annotation of the predicted genes was performed by blasting their sequences against InterPro (https://www.ebi.ac.uk/interpro/), KEGG (http://www.genome.jp/kegg/), Swissprot (http://www.uniprot.org/) and TREMBL (http://www.uniprot.org/) database with an e-value cutoff of 1e^-5^. BUSCO was then used to assess completeness of the final genome assembly[^9^](#_ENREF_9). The non-coding RNA were identified by searching the genome assembly against the Rfam11.0 database using INFERNAL (v1.1) with default parameters (http://infernal.janelia.org/). tRNA was identified by using tRNAscan-SE (<http://lowelab.ucsc.edu/tRNAscan-SE/>).

**Supplemental Method 3. Genome evolution**

To study the evolution of the *Salvia miltiorrhiza*, the first step is to identify orthologous genes from selected closely related species genomes and to further get the *Salvia miltiorrhiza* specific gene families. Proteins from all selected closely species were performed all-by-all BLASTP. Then, OrhtoMCL (v2.0.2)[^10^](#_ENREF_10) was used to cluster the similarities gene pairs into groups. All single copy orthologous gene clusters were extracted and used to construct the phylogenetic tree based on the Maximum Likelihood method by PhyML (v3.0)[^11^](#_ENREF_11) software package. The Bayesian Relaxed Molecular Clock (BRMC) method was utilized to estimate the divergence time of species by MCMCTREE (v4.4), a part of PAML package. ‘Correlated molecular clock’ and ‘JC69’ models were selected. The MCMC process of PAML MCMCTREE program[^12^](#_ENREF_12) was used 100,000 times during species with sample frequency of 2 and 10,000 iterations. Published divergence time between *S.tuberosum* - *C.annuum* was 7.2 to 7.4 Mya[^13^](#_ENREF_13) was used to calibrate the divergence time.

**Supplementary References**

1. Zou CS, et al. A high-quality genome assembly of quinoa provides insights into the molecular basis of salt bladder-based salinity tolerance and the exceptional nutritional value. Cell Res 27, 1327-1340 (2017).

2. Benson G. Tandem repeats finder: a program to analyze DNA sequences. Nucleic acids research 27, 573-580 (1999).

3. Edgar RC, Myers EW. PILER: identification and classification of genomic repeats. Bioinformatics 21 Suppl 1, i152-158 (2005).

4. Xu Z, Wang H. LTR_FINDER: an efficient tool for the prediction of full-length LTR retrotransposons. Nucleic acids research 35, W265-268 (2007).

5. Birney E, Clamp M, Durbin R. GeneWise and Genomewise. Genome research 14, 988-995 (2004).

6. Elsik CG, Mackey AJ, Reese JT, Milshina NV, Roos DS, Weinstock GM. Creating a honey bee consensus gene set. Genome biology 8, R13 (2007).

7. Trapnell C, et al. Transcript assembly and quantification by RNA-Seq reveals unannotated transcripts and isoform switching during cell differentiation. Nature biotechnology 28, 511-515 (2010).

8. Kocher SD, et al. The draft genome of a socially polymorphic halictid bee, Lasioglossum albipes. Genome biology 14, R142 (2013).

9. Waterhouse RM, et al. BUSCO Applications from Quality Assessments to Gene Prediction and Phylogenomics. Molecular biology and evolution 35, 543-548 (2018).

10.Li L, Stoeckert CJ, Jr., Roos DS. OrthoMCL: identification of ortholog groups for eukaryotic genomes. Genome research 13, 2178-2189 (2003).

11.Guindon S, Gascuel O. A simple, fast, and accurate algorithm to estimate large phylogenies by maximum likelihood. Systematic biology 52, 696-704 (2003).

12.Yang Z. PAML 4: phylogenetic analysis by maximum likelihood. Molecular biology and evolution 24, 1586-1591 (2007).

13.Li F, et al. Genome sequence of the cultivated cotton Gossypium arboreum. Nature genetics 46, 567-572 (2014).
