# Supplementary material for: Expansion within the CYP71D subfamily drives the heterocyclization of tanshinones synthesis in Salvia miltiorrhiza
Source: Nat Commun. 2021 Jan 29;12:685. doi: 10.1038/s41467-021-20959-1 (PMC7846762; doi:10.1038/s41467-021-20959-1)
Supplement: Supplementary file 3 — Reporting Summary [file 41467_2021_20959_MOESM3_ESM.pdf]

nature research

Corresponding author(s): Juan Guo
Guanghong Cui
Xiaoquan Qi
Luqi Huang

Last updated by author(s): Dec 17, 2020

Reporting Summary

Nature Research wishes to improve the reproducibility of the work that we publish. This form provides structure for consistency and transparency in reporting. For further information on Nature Research policies, see our Editorial Policies and the Editorial Policy Checklist.

Statistics

For all statistical analyses, confirm that the following items are present in the figure legend, table legend, main text, or Methods section.

n/a	Confirmed	
☐	☒	The exact sample size (*n*) for each experimental group/condition, given as a discrete number and unit of measurement
☐	☒	A statement on whether measurements were taken from distinct samples or whether the same sample was measured repeatedly
☐	☒	The statistical test(s) used AND whether they are one- or two-sided<br>*Only common tests should be described solely by name; describe more complex techniques in the Methods section.*
☐	☒	A description of all covariates tested
☐	☒	A description of any assumptions or corrections, such as tests of normality and adjustment for multiple comparisons
☐	☒	A full description of the statistical parameters including central tendency (e.g. means) or other basic estimates (e.g. regression coefficient) AND variation (e.g. standard deviation) or associated estimates of uncertainty (e.g. confidence intervals)
☐	☒	For null hypothesis testing, the test statistic (e.g. *F*, *t*, *r*) with confidence intervals, effect sizes, degrees of freedom and *P* value noted<br>*Give P values as exact values whenever suitable.*
☒	☐	For Bayesian analysis, information on the choice of priors and Markov chain Monte Carlo settings
☒	☐	For hierarchical and complex designs, identification of the appropriate level for tests and full reporting of outcomes
☐	☒	Estimates of effect sizes (e.g. Cohen's *d*, Pearson's *r*), indicating how they were calculated

Our web collection on statistics for biologists contains articles on many of the points above.

Software and code

Policy information about availability of computer code

Data collection	No software were used for data collection
Data analysis	Software and Tools used in this article (further details about command lines are found in Methods and/or upplementary Materials file): Falcon (v1.7.4), PyMOL (version1.7.4.0.),OrhtoMCL (v2.0.2), PhyML (v3.0), MCMCTREE (v4.4), CAFE (v2.1), AutoDockTools(version1.5.6), SSPACE (v3.0), GapCloser (v1.12), RepeatMasker (v4.0.3), RepeatModeler (v1.0), LTR-FINDER (v1.05), GLEAN(v1.1), Cufflinks (v2.2.1), LACHESIS (http://shendurelab.github.io/LACHESIS), K-mer Analysis Toolkit (http://www.earlham.ac.uk/kat-tools), HABOT(v2) (https://github.com/asarum/HABOT2), JCVI (https://www.jcvi.org/), RepeatProteinMask (3.3.0) (http://www.repeatmasker.org), GlimmerHMM (v1.1.0) (http://ccb.jhu.edu/software/glimmerhmm/), Augustus (v3.0.2) (http://bioinf.uni-greifswald.de/augustus/), INFERNAL (v1.1)(http://infernal.janelia.org/), tRNAscan-SE (http://lowelab.ucsc.edu/tRNAscan-SE/), LASTAL(http://last.cbrc.jp/), DISCOVAR (https://software.broadinstitute.org/software/discovar/blog/)

For manuscripts utilizing custom algorithms or software that are central to the research but not yet described in published literature, software must be made available to editors and reviewers. We strongly encourage code deposition in a community repository (e.g. GitHub). See the Nature Research guidelines for submitting code & software for further information.

Data

Policy information about availability of data

All manuscripts must include a data availability statement. This statement should provide the following information, where applicable:

- Accession codes, unique identifiers, or web links for publicly available datasets
- A list of figures that have associated raw data
- A description of any restrictions on data availability

The data supporting the findings of this work are available within the paper and the Supplementary Information files. A reporting summary for this article is

available as a Supplementary Information file. The data sets generated and analyzed during this study are available from the corresponding author upon request. The genome sequence data have been deposited under NCBI BioProject number PRJNA682867. The source data underlying Figures 3b, 4b, 8b, Supplementary Figures 4, 12, 14, Supplementary Table 13 and Supplementary Data 3, 4 as well as the information of Chromosome 6 are provided as a Source Data file. The databases of KEGG (http://www.genome.jp/kegg/), Swissprot (http://www.uniprot.org/), REMBL (http://www.uniprot.org/), InterPro Database(https://www.ebi.ac.uk/interpro/) were used in this study.

Field-specific reporting

Please select the one below that is the best fit for your research. If you are not sure, read the appropriate sections before making your selection.

[x] Life sciences [ ] Behavioural & social sciences [ ] Ecological, evolutionary & environmental sciences

For a reference copy of the document with all sections, see nature.com/documents/nr-reporting-summary-flat.pdf

Life sciences study design

All studies must disclose on these points even when the disclosure is negative.

Sample size	The sample size of genome sequencing is "1". For RNA-seq of RNAi and wild type lines are used 3 biological samples, respectively. For compounds determination experiments six biological independent samples from RNAi line and five biological independent samples from wild type line were generated.
Data exclusions	We only excluded sequences that were of low quality for genome assembly.
Replication	For RNA-seq experiments, three biological replicates were generated in both RNAi and wild type lines.<br>For compounds determination experiments, six biological replicates from RNAi line and five biological replicates from wild type line were generated in this study.
Randomization	The explants of Salvia miltiorrhiza used to induce transgenic plants were randomly selected.
Blinding	The explants of Salvia miltiorrhiza were blinded assigned to induce transgenic plants .

Reporting for specific materials, systems and methods

We require information from authors about some types of materials, experimental systems and methods used in many studies. Here, indicate whether each material, system or method listed is relevant to your study. If you are not sure if a list item applies to your research, read the appropriate section before selecting a response.

Materials & experimental systems

n/a	Involved in the study
[x]	[ ] Antibodies
[x]	[ ] Eukaryotic cell lines
[x]	[ ] Palaeontology and archaeology
[x]	[ ] Animals and other organisms
[x]	[ ] Human research participants
[x]	[ ] Clinical data
[x]	[ ] Dual use research of concern

Methods

n/a	Involved in the study
[x]	[ ] ChIP-seq
[x]	[ ] Flow cytometry
[x]	[ ] MRI-based neuroimaging
