# Supplementary material for: Expansion within the CYP71D subfamily drives the heterocyclization of tanshinones synthesis in Salvia miltiorrhiza
Source: Nat Commun. 2021 Jan 29;12:685. doi: 10.1038/s41467-021-20959-1 (PMC7846762; doi:10.1038/s41467-021-20959-1)
Supplement: Supplementary file 4 — Description of Additional Supplementary Files [file 41467_2021_20959_MOESM4_ESM.pdf]

Description of Additional Supplementary Files

Supplementary Data 1

Expanded gene family in Danshen.

Supplementary Data 2

Contracted gene family in Danshen.

Supplementary Data 3

Downregulated diterpenoids indicated by targeted metabolomics analysis of WT and CYP71Ds-RNAi lines by LC-qTOF-MS. Source data are provided as a Source Data file.

Supplementary Data 4

Upregulated diterpenoids indicated by targeted metabolomics analysis of WT and CYP71Ds-RNAi lines by LC-qTOF-MS. Source data are provided as a Source Data file.
