# Supplementary material for: Expansion within the CYP71D subfamily drives the heterocyclization of tanshinones synthesis in Salvia miltiorrhiza
Source: Nat Commun. 2021 Jan 29;12:685. doi: 10.1038/s41467-021-20959-1 (PMC7846762; doi:10.1038/s41467-021-20959-1)
Supplement: Supplementary file 2 — Peer Review File [file 41467_2021_20959_MOESM2_ESM.pdf]

Reviewer #1 (Remarks to the Author):

This is a very well written and comprehensive paper on evolutionary aspect of how Salvia miltiorrhiza via duplications of the CYP71D subfamily evolved to do heterocyclization in tanshinone biosynthesis.

Somehow I find the abstract less interestingly written then paper and it might attract more attention if there were more focus on the evolutionary aspect. Same goes, but to a lesser extend, for the title that focus mainly on the biochemistry.

If would be helpful for the general reader if the CAFÉ software was explained, and in figure 8.a is not clear what kind of tree is represented.

There is a spelling mistake on line 178, found should be round.

Reviewer #2 (Remarks to the Author):

The genome of Danshen is well-known as high heterozygosity and difficult to be assembled. The authors used an selfings line to reduce the heterozygosity of the genome. In this manuscript, they provided an improved draft of the Danshen genome using Illumina, PacBio RSII and Hi-C sequencing technology. The results of genome assembly could be more rich, and the accuracy of genome and annotated genes needs to be further evaluated.
1.Lines 287-288: How do you define the heterozygosity? Please compare it with the published manuscripts and natural hybrid Danshen.
2.Lines 296-298: The assembled genome covered was only 89% of predicted size?
3.Lines 558-562: The Hi-C assembly need to be added to the results of genome assembly. The assessment results of Hi-C assembly genome need to be provided.
4.How do you assess the prediction genes? The assessment results of annotated genes need to be provided.
5.Line 361: The 450bp nucleotide should be compared with all genes to test its specificity.
6.Line 367: What about the consistency of the ten transgenic lines?
7.Line 408: The bold numbers should be changed to chemical name.

Reviewer #3 (Remarks to the Author):

The manuscript by Ma et al. described their efforts on identification and characterization of the key p450s involved in tanshinone biosynthesis, especially the furan ring formation. The authors adopted an interdisciplinary approach, including genome sequencing, transciptome analysis, creation and analysis of transgenic plant lines, expression and biochemical characterization of the proposed genes. Plant biosynthetic studies are very challenging and the work presented here is sufficient and solid. Please take care of the following issues to further improve the quality and impact of the reported work.
1. Line 36-38, “Notably, this consolidated and expanded upon previous findings, revealing a large biosynthetic gene cluster associated with the early steps in tanshinone production.” This sentence is not clear. Please rewrite.
2. Line 45 – 48, ..” found to catalyze not only hydroxylation at carbon-16 (C16), but also 14,16-ether (hetero)cyclization to form the ‘D’-ring with intermediates in which C20 has already been lost.” This sentence needs to be rewritten because too much information are mixed together.
3. Line 67, this sentence needs to be rewritten. “15,16-dihydrotanshinone (2), which exert at least pro-apoptotic activity”
4. Fig 1. Structure and font are too small. In addition, in Fig. 1b, it might be a good idea to show the

structures of the intermediates of the GGPP to miltiradiene steps.

5. Line 89- 91, "the ability of the latter two CYPs to react with more than one plausible intermediate metabolite suggests the potential for at least bifurcation of tanshinone biosynthesis (Fig. 1b)" needs to be rewritten because it is common for CYPs to have relaxed substrate specificity.

6. Line 244-248, "in vitro activity assays were performed on a shaking incubator at 30°C for 4 h in 500 μL of 100 mM Tris-HCl, pH 7.5, containing 0.5 mg total microsomal protein, and 500 μM NADPH, along with a regenerating system (consisting of 5 μM FAD, 5 μM FMN, 5 mM glucose-6-phosphate, 1 unit glucose-6-phosphate dehydrogenase), and 100 μM of substrate." This enzymatic assay condition may need to be double-checked. You have only 100 μM of substrate and you have 500 μM NADPH already. Why do you need the regenerating system? In addition, in the 0.5 mg of the microsomal protein, how much is the desired target enzyme?

7. Line 258 – 259, "system was expanded from 500 uL to 100 mL, with accompanying increase in amount of microsomal protein content. The substrate concentration also was increased to 30 mM," Because the enzymatic reaction conditions listed in item 6 are confusing, you might need to explain what is going on here too. Relative to conditions in item 6, the substrate concentration is now increased by 300-fold.

8. Line 273 – 275, what are the criteria to check the quality of your structural model? In this case, you built the heme cofactor into the structural model, any additional validation to the check the quality?

9. Line 321 – 323, "This analysis indicates that 164 gene families underwent significant expansion and 142 gene families underwent contraction in Danshen (Supplementary Data 1 and 2)." Please be more specific. The 164 gene family expansion and 142 gene familycontraction in Danshen relative to what?

10. Fig. 4: the structure and the font used in the table are way too small. It is difficult to read. In addition, I do not understand what they mean for the "double bands" column.

11. Line 385- 392, you listed up- and down-regulated genes and in the subsequent sections, you did explain the function of some of these genes. It might be better to add a short summary section here to indicate how many of them are directly involved in Tanshinone biosynthesis and how many of them could not be explained at this point.

12. In this manuscript, there are tens of compounds discussed. It will be easier to number all compounds sequentially to make the paper easier to read and follow. When I got into the metabolomic and enzymatic sections, it took me a while to match the discussion with compounds and their structures.

13. Line 441, "Only CYP71D375 reacted with 4, with three products detected (Fig. 5a)." This is not a correct statement. The more appropriate statement is "only CUP71D375 accepts 4 as the substrate, with three products detected"

14. In the "Biochemical analysis of the targeted CYPD71D clade" section, the authors discussed MS and NMR data. Please organized these spectra sequentially based on the compound number used in the manuscript. In addition, some of the NMR spectra are not clean. The compound structural assignments have a great chance to be right. However, to be published on a high profile journal, it is better to have clean spectra and some of the NMR spectra are not high quality either.

15. Line 471, "only CYP71D375 reacted with 6, 471 with two products detected". The same issue, CYP71D375 is the catalyst.

16. Supplementary fig. 7. These are important information for the paper, if you can get NMR data, it will be better to show them instead of just the MS data.

17. Line 481, "However, CYP71D411 reacted with sugiol (3)" The same issue, CYP71D411is a catalyst.

18. Line 489 – 491, "In particular, the results suggest that CYP71D375 and CYP71D373 are important for heterocyclization to form the characteristic 'D'-ring of the tanshinones (Fig. 6a)". Yes, both CYP71D375 and CYP71D373 catalyze the hydroxylation reaction (5 $\rightarrow$9). However, the biochemical data indicates that only CYP71D375 catalyzes the cyclization reaction. If possible, please show the kinetic information, which will provide additional data for accurate functional assignments. It is highly possible that CYP71D373 catalyzes the hydroxylation reaction (5 $\rightarrow$9), while CYP71D375 catalyzes the cyclization. To accurately assign the function, kinetic information will be critical.

19. Line 520 – 550: the structural modeling section. You may delete this section because you have enough data for this manuscript already and this section is basically speculative information and it does not add much value to your manuscript.

20. Fig. 6b, the catalytic mechanisms, please consult mechanisms in a non-heme iron enzyme-catalyzed epoxidation reaction (from alcohol): Nature, 437, 838-844, and Nature, 496, 114 – 118.

Response to Editor and Reviewer for Manuscript <u>NCOMMS-20-27146</u>

Dear Editor and Reviewers,

We appreciate all of your constructive comments and suggestions, and have modified our manuscript accordingly. Below we detail the changes made in response to the specific comments made by the reviewers.

Reviewer 1：

This is a very well written and comprehensive paper on evolutionary aspect of how Salvia miltiorrhiza via duplications of the CYP71D subfamily evolved to do heterocyclization in tanshinone biosynthesis.

Comment 1:

Somehow I find the abstract less interestingly written then paper and it might attract more attention if there were more focus on the evolutionary aspect. Same goes, but to a lesser extend, for the title that focus mainly on the biochemistry.

Response 1:

We appreciate your suggestion. According to the comments of yours and Reviewer 3's, we have rewritten the abstract. Please find it in the manuscript.

Comment 2:

It would be helpful for the general reader if the CAFE software was explained, and in figure 8.a is not clear what kind of tree is represented.

Response 2:

Thank you for your suggestions. We have updated the procedure of using CAFE to explore the expansion and contraction gene families in **Genome evolution** section in

line 183-184 and gave more information about the tree in Figure 8a in the revised manuscript.

<u>Comment 3:</u>

There is a spelling mistake on line 178, found should be round

<u>Response 3:</u>

Thank you very much for your comments. The spelling mistake has been corrected in line 164 in the revised manuscript.

Reviewer 2

The genome of Danshen is well-known as high heterozygosity and difficult to be assembled. The authors used a selfings line to reduce the heterozygosity of the genome. In this manuscript, they provided an improved draft of the Danshen genome using Illumina, PacBio RSII and Hi-C sequencing technology. The results of genome assembly could be more rich, and the accuracy of genome and annotated genes needs to be further evaluated.

Comment 1:

Lines 287-288: How do you define the heterozygosity? Please compare it with the published manuscripts and natural hybrid Danshen.

Response 1:

Thank you very much for your suggestion. The heterozygosity was obtained from 17-mer analysis using 26.73 Gb data (line 272) in this study. While k-mer analysis in the two previously reported Danshen genomes, couldn't give single peak. Thus, they didn't give the heterozygosity from k-mer analysis. Xu et al. reported that there were 2.76 single nucleotide variation per Kb by mapping the Illumina reads onto the draft assembly. In our analysis, it is revealed that the line bh2-7 has a heterozygosity of 0.64 SNVs per Kb, which is 4.3 fold lower than the previous one. The result has been added in line 287-291 in **Sequencing, Assembly and Annotation** section.

Comment 2:

Lines 296-298: The assembled genome covered was only 89% of predicted size?

Response 2:

The predicted Danshen genome size is 623.58 Mb (k-mer analysis) and 622Mb (flow cytometry), while the assembly genome is 557 Mb. The assembled genome covered 89% of predicted size. This may due to the estimated high proportion (71.84%) of repetitive sequences in the Danshen genome. This phenomenon could also be found in previous genome sequence reports about Danshen and other species (Danshen with 87% (538 Mb/615 Mb) (Xu et al., Molecular Plant, 2016), *Macleaya cordata* with 70% (378 Mb/540.5Mb) (Liu et al., Molecular Plant, 2017)).

Comment 3:

Lines 558-562: The Hi-C assembly need to be added to the results of genome assembly. The assessment results of Hi-C assembly genome need to be provided.

Response 3:

Thanks for your suggestion. We'd like to build a chromosome-scale Danshen genome. The assembly parameters of Hi-C result are acceptable (Table 1-2), but the interaction heat-map of chromosomal fragments (below Fig 1) seems not so good. It showed that pseudochromosome 6 and 8 had relative strong interactions within the pseudochromosomes, while other pseudochromosomes didn't attain the expected value. We thus seek to re-sequence the Danshen genome combining the latest technologies including Pacbio, Nanopore and 10X genomic to improve the assembly quality. But the genome-survey results showing neither combination would promote the assembly quality of Danshen genome due to the existence of high proportion repetitive sequences. Thus we did not focus more on the Hi-C assisted genome assembly results.

However, the bad quality of whole genome Hi-C results would not affect the gene clusters identified before with supports listed below.

First, the two regions, ferruginol biosynthetic gene cluster and CYP71D tandem gene array, were located on the terminals of Chr6 with relatively high interactions (as highlighted using asterisks in the updated Supplementary Fig 12).

Second, we performed a syntenic analysis of the above 2 genes clusters with *Scutellaria baicalensis* genome since it has the chromosome-scale assembly results. The ferruginol biosynthetic gene cluster located in scaffold79 and scaffold357. Together with scaffold11, these three scaffold regions (1.41 Mb) formed a well collinear region with parts of Chr06 of *S. baicalensis* (below Figure 2). CYP71D tandem gene array located in scaffold117 and scaffold447 and formed a 0.69 Mb collinear region with *S. baicalensis* (below Figure 3). The strong collinearity with *S. baicalensis* was a solid evidence to support assembly accuracy of at least these regions.

Last, in order to further identify this assembly, we designed PCR primers to amplify the gaps between the scaffolds.

Table 1. Statistical figures of Hi-C assembly

Items	Contig_len(bp)	Contig_num	Scaffold_len(bp)	Scaffold_num
Total	538,158,552	4,076	541,582,483	340
Max	2,184,809	-	79,967,116	-
Number>=2000bp	-	3,576	-	247
N50	413,460	394	73,872,369	4
N60	328,169	539	69,328,290	5
N70	268,582	721	65,455,062	6
N80	195,817	954	65,455,062	6
N90	119,340	1,304	51,567,629	7

Table 2. Scaffold number and length grouped on pseudochromosomes

Pseudomolecule	Scaffold Num	Length
chr1	149	79,967,116
chr2	190	77,701,906
chr3	149	74,021,812
chr4	143	73,872,369
chr5	125	69,328,290
chr6	96	65,455,062
chr7	113	51,567,629
chr8	74	45,078,130
Total anchored	1,039	536,992,314
unanchored	332	4,590,169

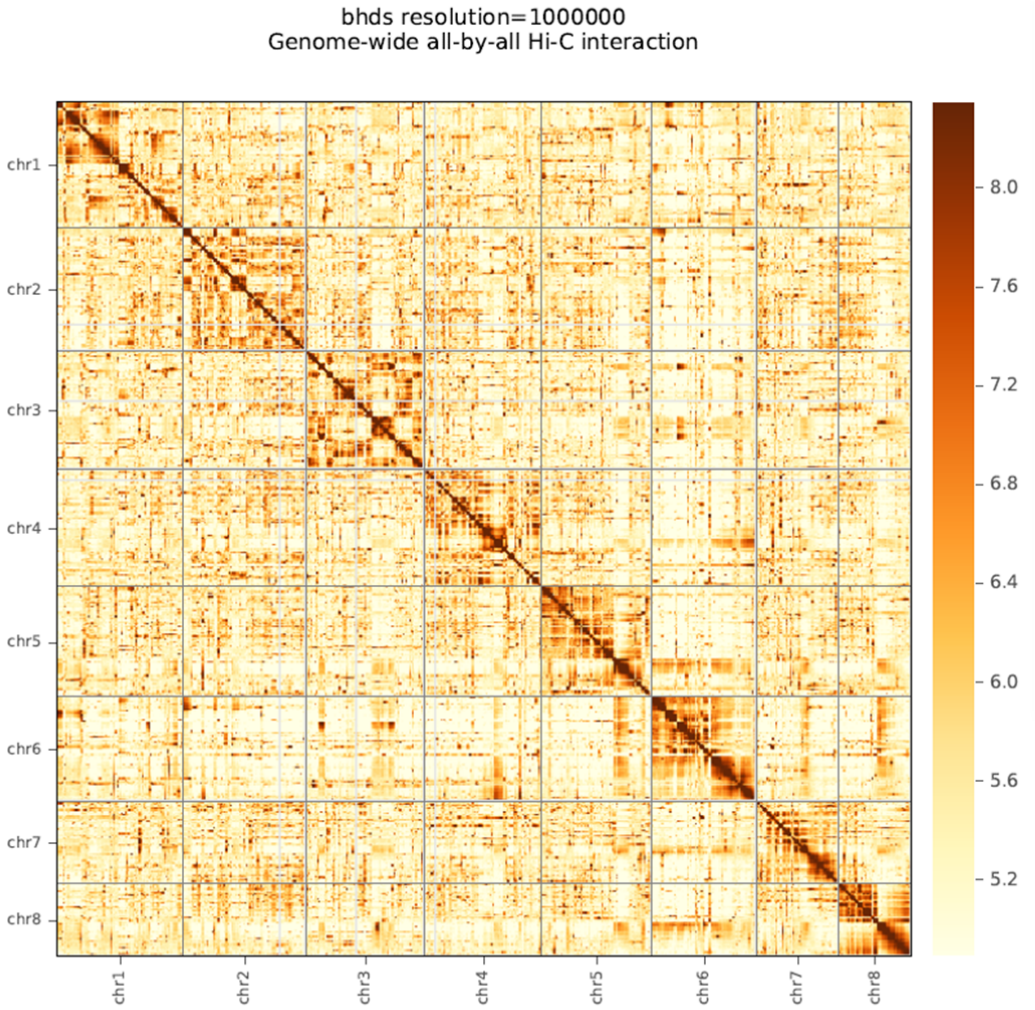

Figure 1. Interaction heat-map of chromosomal fragments based on Hi-C analysis. chr1-chr8 indicate Lachesis Groups 1-8.

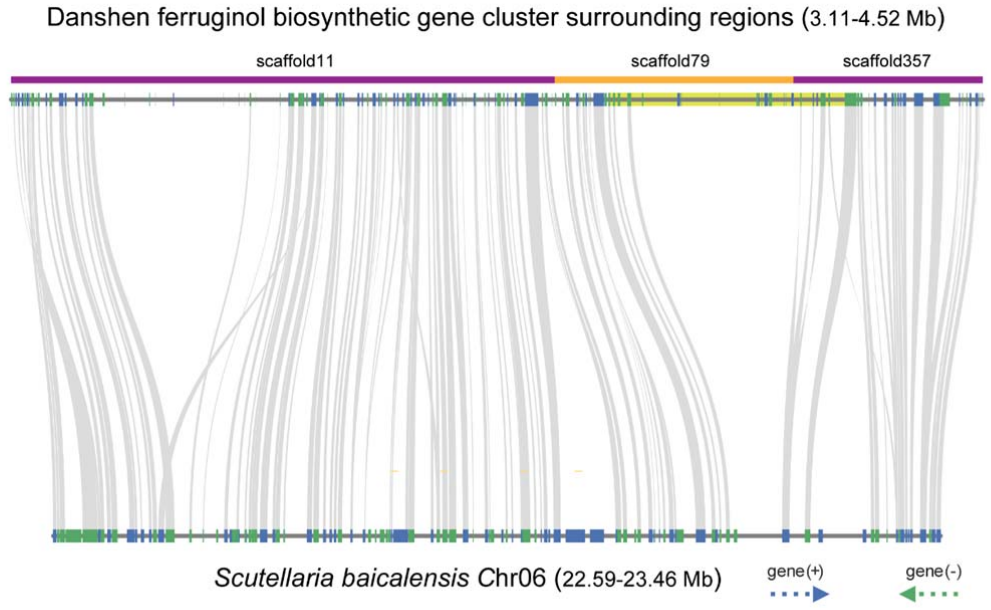

Figure 2. Syntenic analysis of ferruginol biosynthetic gene cluster with *S. baicalensis*. The ferruginol biosynthetic gene cluster was highlighted in yellow.

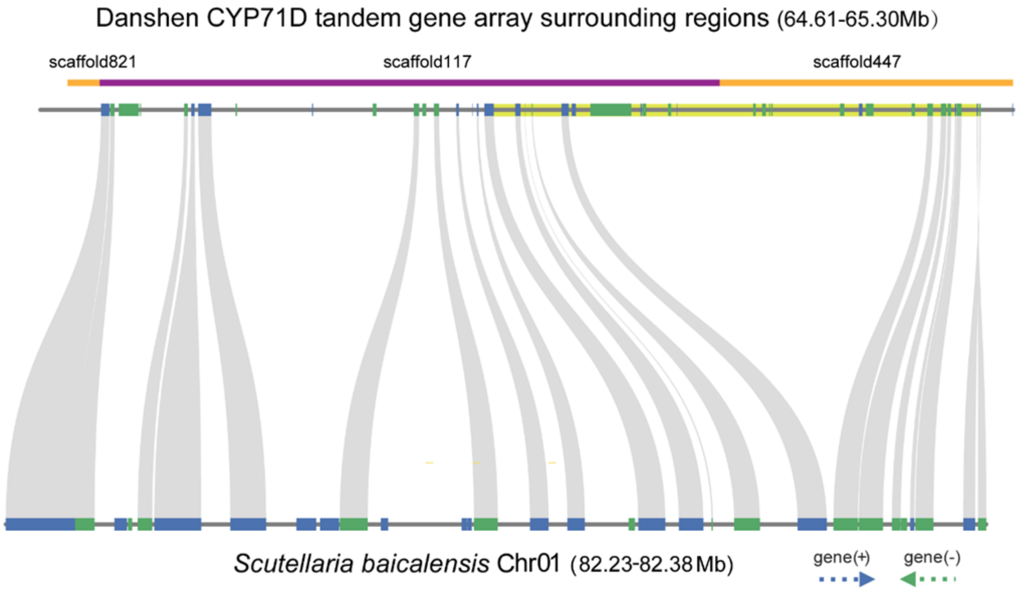

Figure 3. Syntenic analysis of CYP71D tandem gene array with *S. baicalensis*. The CYP71D tandem gene array was highlighted in yellow.

<u>Comment 4:</u>

How do you assess the prediction genes? The assessment results of annotated genes need to be provided.

<u>Response 4:</u>

Thanks for your suggestion. We add these data in line 294-298 in **Sequencing, Assembly and Annotation** section. First, BUSCO and ESTs from transcriptome sequencing analysis indicated 91.10% and 99.56% completeness, respectively. Second, annotated genes were compared with two previously predicted gene sets of Danshen genome. The number of annotated genes was similar to the two previously reported genome, 30,478 and 34,598, individually. Last, about 81.97% of the genes have homologs in the TrEMBL protein database, and 67.83% can be classified by InterPro. In summary, 83.13% genes have either known homologs or can be functionally classified.

<u>Comment 5:</u>

Line 361: The 450bp nucleotide should be compared with all genes to test its specificity.

<u>Response 5:</u>

Thank you for your suggestion. We did global blast against the whole genome to reinforce the specificity of these 453 bp nucleotides. Supplemental table 13 was added in the revised manuscript.

<u>Comment 6:</u>

Line 367: What about the consistency of the ten transgenic lines?

<u>Response 6:</u>

The ten *CYP71Ds*-RNAi plants exhibited a distinct orange phenotype in comparison with the wild-type root and there were no other obvious phenotypic differences. The replication information was showed in Reporting Summary. For RNA-seq experiments, three biological replicates were generated in both RNAi and wild type lines. For compounds determination experiments, six biological replicates from RNAi

line and five biological replicates from wild type line were generated in this study. According to the SE value showed in Figure 4b, Supplementary Data 3 and 4, there is well consistency of these transgenic lines.

Comment 7:

Line 408: The bold numbers should be changed to chemical name.

Response 7:

Thank you very much for your comment. The compounds were changed to chemical names.

Reviewer 3

The manuscript by Ma et al. described their efforts on identification and characterization of the key p450s involved in tanshinone biosynthesis, especially the furan ring formation. The authors adopted an interdisciplinary approach, including genome sequencing, transciptome analysis, creation and analysis of transgenic plant lines, expression and biochemical characterization of the proposed genes. Plant biosynthetic studies are very challenging and the work presented here is sufficient and solid. Please take care of the following issues to further improve the quality and impact of the reported work.

Comment 1:

Line 36-38, "Notably, this consolidated and expanded upon previous findings, revealing a large biosynthetic gene cluster associated with the early steps in tanshinone production." This sentence is not clear. Please rewrite.

Response 1:

We appreciate your suggestion. According to the comments of yours and the other reviewer's, we have rewritten the abstract. Please find it in the manuscript.

Comment 2:

Line 45 – 48, .." found to catalyze not only hydroxylation at carbon-16 (C16), but also 14,16-ether (hetero)cyclization to form the 'D'-ring with intermediates in which C20 has already been lost." This sentence needs to be rewritten because too much information are mixed together.

Response 2:

We appreciate your suggestion. According to the comments of yours and the other reviewer's, we have rewritten the abstract. Please find it in the manuscript.

Comment 3：

Line 67, this sentence needs to be rewritten. “15,16-dihydrotanshinone (2), which exert at least pro-apoptotic activity”

Response 3:

Thank you very much for your suggestion. We have rewritten the sentence as follow, and highlighted in red in the manuscript in lines 55-57. “However, the tanshinsones are more uniquely characterized by the presence of a 14,16-ether ‘D’-ring. While this heterocycle is typically further oxidized to form a furan, even those with a reduced ‘D’-ring, are termed tanshinones – e.g., cryptotanshinone (**1**) and 15,16-dihydrotanshinone (**2**), which have been reported to exert pro-apoptotic activity.”

Comment 4:

Fig 1. Structure and font are too small. In addition, in Fig. 1b, it might be a good idea to show the structures of the intermediates of the GGPP to miltiradiene steps.

Response 4:

We appreciate your suggestion. The structure and font in Fig. 1 were revised and the steps from GGPP to miltiradiene were added in Fig. 1b.

Comment 5:

Line 89- 91, “the ability of the latter two CYPs to react with more than one plausible intermediate metabolite suggests the potential for at least bifurcation of tanshinone biosynthesis (Fig. 1b)” needs to be rewritten because it is common for CYPs to have relaxed substrate specificity.

Response 5:

Thank you very much for your suggestion. We have revised the sentence as follow, and highlighted it in red in the manuscript in line 78-79. “The substantial promiscuity of CYPs resulted in metabolic network of tanshinone biosynthesis.”

Comment 6：

Line 244-248, "in vitro activity assays were performed on a shaking incubator at 30°C for 4 h in 500 μL of 100 mM Tris-HCl, pH 7.5, containing 0.5 mg total microsomal protein, and 500 μM NADPH, along with a regenerating system (consisting of 5 μM FAD, 5 μM FMN, 5 mM glucose-6-phosphate, 1 unit glucose-6-phosphate dehydrogenase), and 100 μM of substrate." This enzymatic assay condition may need to be double-checked. You have only 100 μM of substrate and you have 500 μM NADPH already. Why do you need the regenerating system? In addition, in the 0.5 mg of the microsomal protein, how much is the desired target enzyme?

Response:6:

Thank you very much for your comment. In the in vitro activity assays, it also worked without regenerating system. We used regenerating system just because sometimes we wanted to make sure for sufficient supply of NADPH in long term or large volume assay. Calculated the desired target enzyme accurately is not always worked as heterologous expression of CYP expressed in *Saccharomyces cerevisiae* is low and the quantitative method for CYPs using carbon monoxide-difference spectrum analysis is sometime in low efficiency. We tried to calculated the amount of target CYPs, however, we are sorry that we did not get an obvious peak in 450nm in carbon monoxide-difference spectrum analysis.

Comment 7：

Line 258 – 259, "system was expanded from 500 uL to 100 mL, with accompanying increase in amount of microsomal protein content. The substrate concentration also was increased to 30 mM," Because the enzymatic reaction conditions listed in item 6 are confusing, you might need to explain what is going on here too. Relative to conditions in item 6, the substrate concentration is now increased by 300-fold.

Response:7:

Thank you very much for your comments, we made a mistake about the concentration here. In order to get enough product for NMR analysis, we enlarged the reaction system. As the reaction system has been expanded from 500 uL to 100 mL, we expected to get as many products as possible. We increased the concentration of the substrate to ensure the substrate was enough. Actually, this is not necessary and we deleted it in the manuscript.

Comment 8：

Line 273 – 275, what are the criteria to check the quality of your structural model? In this case, you built the heme cofactor into the structural model, any additional validation to the check the quality?

Response:8:

Thank you very much for you comment. CYP71D373, CYP71D375, CYP71D411 and CYP71D464 were modeled using SwissModel, with the structure of the most closely related CYP76AH1 (5YLW) serving as the template. The seq identity of CYP71Ds with 5YLW were higher than 30%. The GMQE were 0.65, 0.67, 0.66 and 0.74 which were in the credibility range of 0-1. The QMEAN with -2.74, -2.43, -2.62 and -1.82 were in the range of - 4 to 0, which indicated well matching degree of CYP71Ds structural models with the template, and highly credibility of the modeling. In addition, when heme was built into the structural model, the quality of the structure model was evaluated using ProSA and the ProSA energy graphs were all in a negative range with the calculate Z-score (Figure 4). The model of CYP71Ds with heme were further analyzed with verify 3D and ERRAT, the result showed that over 80% of the residues have averaged 3D-1D score≥0.2, and the overall quality factor calculated with ERRAT were at least 88.913 which showed that the quality of the structural model is reliable for further analysis.

Since the reviewer suggested to delete this section (Comment 19), the validation method and result were not added in manuscript or supplementary figures for now. The figures of Z-score, verify 3D and ERRAT validation could be added if necessary.

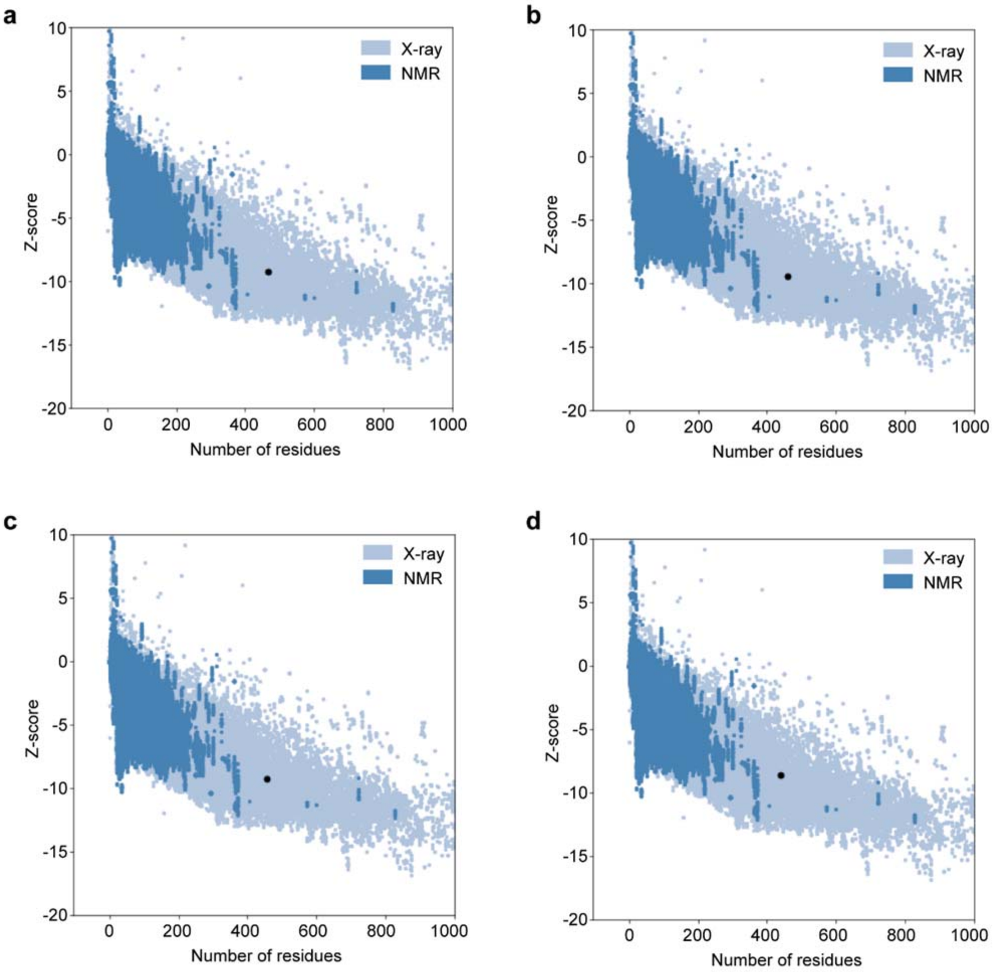

Figure 4. The ProSA energy graphs for (**a**) CYP71D373=-9.26, (**b**) CYP71D375=-9.4, (**c**) CYP71D411=-9.27 and (**d**) CYP71D464=-8.59, respectively.

<u>Comment 9：</u>

Line 321 – 323, “This analysis indicates that 164 gene families underwent significant expansion and 142 gene families underwent contraction in Danshen (Supplementary Data 1 and 2).” Please be more specific. The 164 gene family expansion and 142 gene family contraction in Danshen relative to what?

<u>Response:9:</u>

Thank you very much for your suggestion. The expansion families related to CYPs, acyltransferases, laccases, auxin response factors, genes supposed to in biosynthesis

of salvianolic acid (such as cinnamate 4-hydroxylase, rosmarinic acid synthase, 4-coumarate--CoA ligase), and so on. The contraction families related to cellulose synthase, trehalose 6-phosphate synthase, light-harvesting complex II chlorophyll a/b binding protein, histone superfamily protein, germacrene C synthase, terpene synthase assigned to monoterpenoid biosynthesis, and so on (Supplementary Data 1 and 2). We have added a brief description about the expansion gene families (highlighted in red) in line 314-317 in **Expansion of a clade within the CYP71D subfamily** section .

Comment 10：

Fig. 4: the structure and the font used in the table are way too small. It is difficult to read. In addition, I do not understand what they mean for the "double bands" column.

Response 10:

We appreciate your suggestion. The structure and font in Fig.4 were enlarged. The "double bonds" column means the carbon sites in the structure are connected by double bonds and the spelling mistakes in Fig. 4 c-d were corrected.

Comment 11:

Line 385- 392, you listed up- and down-regulated genes and in the subsequent sections, you did explain the function of some of these genes. It might be better to add a short summary section here to indicate how many of them are directly involved in Tanshinone biosynthesis and how many of them could not be explained at this point.

Response 11:

Thank you very much for your comments. Among these up- and down-regulated genes, the mentioned CPS1, KSL1, CYP76AH1, CYP76AH3, and CYP76AK1 have been characterized involving in biosynthesis of tanshinones. While other genes, except the three characterized CYP71Ds in this study, none of them were functionally characterized.

Comment 12:

In this manuscript, there are tens of compounds discussed. It will be easier to number all compounds sequentially to make the paper easier to read and follow. When I got into the metabolomic and enzymatic sections, it took me a while to match the discussion with compounds and their structures.

Response 12:

In the manuscript, too many compounds were referred to. It is confused. We apologized for that. Actually, the compounds involved in catalytic steps were now numbered in order of their appearance. But those compounds not included in the catalytic steps were not numbered. The compounds in Fig. 6 were also changed to show with numbers to make it easier to follow.

Comment 13:

Line 441, "Only CYP71D375 reacted with 4, with three products detected (Fig. 5a)." This is not a correct statement. The more appropriate statement is "only CYP71D375 accepts 4 as the substrate, with three products detected"

Response 13:

Thank you very much for your comment. We have revised the description in line 437 which highlighted in red.

Comment 14 : .

In the "Biochemical analysis of the targeted CYPD71D clade" section, the authors discussed MS and NMR data. Please organized these spectra sequentially based on the compound number used in the manuscript. In addition, some of the NMR spectra are not clean. The compound structural assignments have a great chance to be right. However, to be published on a high profile journal, it is better to have clean spectra and some of the NMR spectra are not high quality either.

Response :14:

Thank you very much for your comment. Product **7** and **12** were extracted and purified again, the NMR spectrum were reanalyzed and showed in Supplementary Fig. 6 and 9.

Comment 15:

Line 471, "only CYP71D375 reacted with 6, 471 with two products detected". The same issue, CYP71D375 is the catalyst.

Response 15:

Thank you very much for your comment. We have revised the description in line 466 which highlighted in red.

Comment 16:

Supplementary fig. 7. These are important information for the paper, if you can get NMR data, it will be better to show them instead of just the MS data.

Response 16:

Thank you very much for your comment. Pure product substrate **6** is hard to obtained by separation in plant, while we still tried to isolate **6** from plant and used as substrate to produce **10** by catalytic method, however, the purity and yield were still not enough for NMR identification, we feel really sorry for that. The standard compound of **11** was purchased recently and the structure of **11** was confirmed comparing to the standard (Supplementary fig. 7d).

Comment 17:

Line 481, "However, CYP71D411 reacted with sugiol (3)" The same issue, CYP71D411is a catalyst.

Response 17:

Thank you very much for your comment. We have revised the description in line 476 which highlighted in red.

Comment 18:

Line 489 – 491, “In particular, the results suggest that CYP71D375 and CYP71D373 are important for heterocyclization to form the characteristic ‘D’-ring of the tanshinones (Fig. 6a)”. Yes, both CYP71D375 and CYP71D373 catalyze the hydroxylation reaction (5 →9). However, the biochemical data indicates that only CYP71D375 catalyzes the cyclization reaction. If possible, please show the kinetic information, which will provide additional data for accurate functional assignments. It is highly possible that CYP71D373 catalyzes the hydroxylation reaction (5 →9), while CYP71D375 catalyzes the cyclization. To accurately assign the function, kinetic information will be critical.

Response 18:

We appreciate your suggestion. We tried to calculated the kinetic parameters, however, heterologous expression of this two CYPs in *Saccharomyces cerevisiae* is low and sometimes the quantitative method for CYPs using CO did not work well. In addition, sequentially multi-step catalytic of these CYPs resulted in kinetic analysis rather confused. However, we are sure that it is CYP71D375 catalyzing compound **4** to produce **1**, catalyzing compound **5** to produce **2** and catalyzing compound **6** to produce **11**. CYP71D373 catalyzed compound **5** to produce **2**. Because the four in vitro assays were analyzed independently using empty vector as control.

Comment 19:

Line 520 – 550: the structural modeling section. You may delete this section because you have enough data for this manuscript already and this section is basically speculative information and it does not add much value to your manuscript.

Response 19:

Thank you very much for your suggestion. This section is a supplement for verified the key site of CYP. If the reviewers and editors think it is unnecessary. We could delete this section.

Comment 20:

Fig. 6b, the catalytic mechanisms, please consult mechanisms in a non-heme iron enzyme-catalyzed epoxidation reaction (from alcohol): Nature, 437, 838-844, and Nature, 496, 114 – 118.

Response 20:

While we appreciate the suggestion, we respectfully point out that the mechanisms proposed in these publications apply to non-heme iron dioxygenases, while those here must apply to heme-iron containing cytochromes P450. This then rules out the bidentate substrate coordination of iron proposed for the dioxygenase mechanism. Accordingly, we are forced to use the originally proposed mechanism, although we have corrected the original description (see red text in line 498).

We hope you find the revised manuscript acceptable.

With best regards,

Juan Guo

Reviewer #1 (Remarks to the Author):

I find that the revised version has satisfactory addressed the comments raised by the reviewers.

Reviewer #2 (Remarks to the Author):

The authors had answered my questions.

Reviewer #3 (Remarks to the Author):

The authors did a nice job addressing my previous comments. I still have reservation on the proposed reaction mechanism. At this point, it is a starting point. Someone in the future may be able to characterize the mechanistic details. Therefore, it might be fine to publish at its current format.
